# Occurrence of caffeoylquinic acids in bamboo suspension cells cultured under light

**DOI:** 10.5511/plantbiotechnology.24.0812a

**Published:** 2024-12-25

**Authors:** Naoki Ube, Yasuo Kato, Taiji Nomura

**Affiliations:** 1 Biotechnology Research Center and Department of Biotechnology, Toyama Prefectural University, 5180 Kurokawa, Imizu, Toyama 939-0398, Japan

**Keywords:** bamboo cells, chlorogenic acids, hydroxycinnamoylquinic acids, light-induced secondary metabolites, *Phyllostachys nigra*

## Abstract

Rational metabolic-flow switching is an effective strategy that we previously proposed to produce exogenous high-value natural products in cultured plant cells through redirecting a highly active inherent metabolic pathway to a pathway producing related exogenous compounds. In previous proof-of-concept studies, we demonstrated that bamboo (*Phyllostachys nigra*; Pn) cells are a suitable host for production of phenylpropanoid-derived compounds, in particular those derived from feruloyl- and *p*-coumaroyl-CoAs. To expand the utility of Pn cells for production of exogenous metabolites via the rational metabolic-flow switching strategy, it is important to evaluate the metabolic potential of Pn cells under diverse culture conditions. In this study, we examined highly active metabolic pathway(s) in Pn suspension cells cultured under light. The Pn suspension cells strongly accumulated two light-induced compounds. These compounds were isolated and identified as 3-*O*-caffeoylquinic acid (neochlorogenic acid) and its regioisomer 5-*O*-caffeoylquinic acid (chlorogenic acid). Through optimization of the culture conditions, production titers of 3-*O*-caffeoylquinic acid and 5-*O*-caffeoylquinic acid in the Pn suspension cells reached 121 and 77.9 mg l^−1^, respectively. These findings indicate that Pn cells are a suitable host for bioproduction of exogenous metabolites, in particular those derived from caffeoyl-CoA via the rational metabolic-flow switching strategy.

## Introduction

Plants are a source of diverse secondary metabolites potentially useful as raw materials for the production of pharmaceuticals, flavoring agents, and fragrances. Low productivity and a prolonged growth period often limit the commercial production of secondary metabolites using harvested plants. To overcome these limitations, several secondary metabolites have been commercially produced using plant cell culture systems (Wilson and Roberts 2012).

We previously developed a “rational metabolic-flow switching” strategy for production of “exogenous” secondary metabolites using cultured plant cells (Nomura et al. 2018); “exogenous” metabolite denotes the compound that is not inherently present in the wild-type host cells and is produced in the transgenic cells expressing biosynthetic gene(s) from other organisms. As the first step of this procedure, a highly active metabolic pathway(s) of interest in the plant cells is predicted by identifying the major metabolite(s) in the cells. Next, cells are transformed to introduce the exogenous gene(s) encoding the enzyme(s) required to switch the inherent active metabolic flow to the biosynthesis of the exogenous target compound(s). Bamboo (*Phyllostachys nigra*; hereafter Pn) cells were used to demonstrate the effectiveness of this strategy, because they proliferate efficiently in liquid medium and accumulate substantial amounts of phenylpropanoid-derived metabolites, such as lignin and hydroxycinnamoylputrescines (i.e., feruloylputrescine [FP] and *p*-coumaroylputrescine [pCP]), depending on the culture conditions (Nomura et al. 2013; Ogita 2005; Ogita et al. 2012). We previously transformed Pn cells to stably express the barley (*Hordeum vulgare*) agmatine coumaroyltransferase gene (*HvACT*) (Nomura et al. 2018), the *Bacillus amyloliquefaciens* phenolic acid decarboxylase gene (*BaPAD*) (Kitaoka et al. 2021), and the *Pseudomonas putida* KT2440 4-hydroxycinnamoyl-CoA hydratase/lyase gene (*PpHCHL*) (Kitaoka et al. 2020; Ube et al. 2024). The HvACT-transformed Pn cells efficiently produced *p*-coumaroylagmatine and feruloylagmatine. The BaPAD-transformed Pn cells produced primeverose conjugates of 4-vinylphenol and 4-vinylguaiacol. The PpHCHL-transformed Pn cells produced mono- and/or di-glucose conjugates of 4-hydroxybenzoic acid and vanillic acid. These three proof-of-concept studies demonstrated the efficacy of the rational metabolic-flow switching strategy for the production of exogenous metabolites, in addition to the suitability of Pn cells for producing phenylpropanoid-derived metabolites, in particular those derived from feruloyl- and *p*-coumaroyl-CoAs.

To broaden the utility of Pn cells for the production of exogenous metabolites via the rational metabolic-flow switching strategy, it is important to evaluate the metabolic potential of Pn cells under diverse culture conditions. Given that plants, including cell suspensions, can biosynthesize various secondary metabolites in response to abiotic and biotic stresses, Pn cells may also have metabolic potential other than the biosynthesis of pCP and FP under certain culture conditions. However, stress-induced metabolites in Pn cells have not been explored previously. In the present study, we assessed the effects of light treatment on the metabolic changes in Pn suspension cells, because plant cells cultured under light usually show metabolic properties different from those of cells cultured in the dark (Murthy et al. 2014). We observed the accumulation of two major compounds in response to the light treatment of Pn cells. The light-induced compounds were isolated and their structures were elucidated. In addition, the culture conditions were optimized to increase the production levels of the light-induced compounds in Pn cells. Based on the results, the potential contribution of this metabolic response of Pn cells to expanding the utility of the rational metabolic-flow switching strategy is discussed.

## Materials and methods

### Plant materials and culture conditions

The Pn suspension cells, which are currently available from the RIKEN BioResource Research Center (no. rpc00047; https://web.brc.riken.jp/ (Accessed May 16, 2024)), were maintained in modified Murashige and Skoog (MS, Supplementary Table S1) liquid medium supplemented with 680 mg l^−1^ KH_2_PO_4_, 10 µM 4-amino-3,5,6-trichloropyridine-2-carboxylic acid (picloram), and 3% (w/v) sucrose (Murashige and Skoog 1962; Ogita 2005; Ogita et al. 2011). This medium strongly promotes the proliferation of Pn cells. The cells were subcultured in 100 ml liquid medium in a 300 ml Erlenmeyer flask and maintained on a rotary shaker (100 rpm) in darkness at 25°C. The cells were subcultured every 2 weeks by adjusting the initial sedimented cell volume (SCV) to 5% as previously described (Ogita et al. 2011).

The subcultured Pn suspension cells were transferred to the following fresh media with an initial SCV of 5%: MS, Gamborg’s B5 (hereafter B5), and White media (Gamborg et al. 1968; White 1963, Supplementary Table S1). The cells were cultured in 30 ml liquid medium in a 100 ml Erlenmeyer flask on a rotary shaker (100 rpm) under light (light quality, white; light intensity, 80 µmol m^−2^ s^−1^; light/dark (LD) cycle, continuous light) at 25°C for 30 days. The light quality was changed by using different light sources as follows: white (06BL4), red (07CT, λ_max_=660 nm), and blue (06M5, λ_max_=440 nm) (Nippon Medical & Chemical Instruments Co., Ltd., Osaka, Japan). The light intensity and the LD cycle were controlled by the controller of the incubator (Bio Multi Incubator LH-30-8CT, Nippon Medical & Chemical Instruments Co., Ltd.). The light intensity was measured with a photometer (Memory Sensor MES-101, Koito Manufacturing Co., Ltd., Tokyo, Japan). The cells were collected every 5 days as previously described (Nomura et al. 2013) and stored at −30°C until use.

### Extraction and HPLC analysis

To analyze the Pn suspension cell metabolites, cell extracts were prepared by sonication (10 min) using 10 volumes of MeOH containing 2% (v/v) AcOH. After the extracts were centrifuged (21,500×g, 10 min, 4°C), the supernatants were analyzed using a reversed-phase HPLC system (column, TSKgel ODS-100V, 5 µm, 4.6×150 mm, Tosoh, Tokyo, Japan; solvent, 20% (v/v) MeOH containing 0.1% (v/v) trifluoroacetic acid; flow rate, 0.8 ml min^−1^; detection wavelength, 280 nm; and column temperature, 40°C).

### Isolation and structural elucidation of light-induced compounds **1** and **2**

The Pn suspension cells were cultured for 15 days in 100 ml B5 liquid medium in a 300 ml Erlenmeyer flask on a rotary shaker (100 rpm) at 25°C under the light conditions as described in the “Plant materials and culture conditions” section. The cells were collected from 1.5 l liquid culture (15 flasks containing 100 ml culture) on filter paper by vacuum filtration. The collected cells (95 g fresh weight, FW) were extracted with 1 l of 50% (v/v) MeOH containing 2% (v/v) AcOH under sonication for 30 min at room temperature. The resulting extract was filtered through a diatomite pad (Radiolite #3000; Showa Chemical Industry, Tokyo, Japan). The filtrate was concentrated to approximately 100 ml and washed three times with *n*-hexane. The aqueous layer was concentrated and applied to a weak cation-exchange column (WK-40, Mitsubishi Chemical Corp., Tokyo, Japan; 2.5×10 cm; 50 ml) equilibrated with water. For the elution of compounds, water (250 ml) was added to the column. The flow-through and water fractions were combined, concentrated to approximately 20 ml, and applied to an octadecylsilyl (ODS) column (Cosmosil 75C_18_-OPN, Nacalai Tesque, Kyoto, Japan; 3×15 cm; 100 ml) equilibrated with water containing 0.1% (v/v) AcOH. For the sequential elution of compounds, 0%, 5%, 10%, 15%, 20%, 30%, 40%, 50%, and 100% (v/v) MeOH containing 0.1% (v/v) AcOH (300 ml per solvent) were added to the column. The 5% and 10% fractions, containing compound **1**, were combined, concentrated to approximately 5 ml, passed through a membrane filter (Millex-HV, 0.45 µm; Merck, Darmstadt, Germany), and subjected to reversed-phase preparative HPLC (column, TSKgel ODS-80Ts, 5 µm, 20×250 mm, Tosoh; solvent, 28% (v/v) MeOH containing 0.1% (v/v) trifluoroacetic acid; flow rate, 5 ml min^−1^; detection wavelength, 280 nm). The collected fraction was concentrated and lyophilized to obtain 108 mg of compound **1** as a white powder. The 15% and 20% fractions from ODS column chromatography, containing compound **2**, were combined, concentrated, filtered, and subjected to reversed-phase preparative HPLC under the same conditions as described above except that the solvent was 30% (v/v) MeOH containing 0.1% (v/v) trifluoroacetic acid. The collected fraction was concentrated and applied to a solid-phase extraction cartridge (Bond Elut Jr-C18, 1 g, Agilent, Santa Clara, CA, USA) equilibrated with water containing 0.1% (v/v) formic acid. For the sequential elution of compounds, 0%, 5%, 10%, 15%, 20%, and 50% (v/v) MeOH containing 0.1% (v/v) formic acid (10 ml per solvent) was added to the column. The 5% and 10% fractions, containing compound **2**, were combined, concentrated, and lyophilized to obtain 21.0 mg of compound **2** as a white powder.

The NMR spectra of compounds **1** and **2** were recorded in CD_3_OD using the AVANCE 400 spectrometer (Bruker, Karlsruhe, Germany). High-resolution electrospray ionization time-of-flight mass spectrometry (HR-ESI-TOF-MS) analysis was performed using the micrOTOF focus spectrometer (Bruker). The UV spectrum and specific optical rotation were measured using the UV-1800 spectrophotometer (Shimadzu, Kyoto, Japan) and the P-1030 Polarimeter (Jasco, Tokyo, Japan), respectively.

The spectral properties of 3-*O*-caffeoylquinic acid (3-CafQA, **1**) were as follows: HR-ESI-TOF-MS (Supplementary Figure S1A) (positive) *m*/*z* 355.1022 [M+H]^+^ (calcd. for C_16_H_19_O_9_^+^, 355.1024); UV (MeOH) λ_max_ (logε) 219 nm (4.18), 245 nm (4.00), 330 nm (4.18); [α]_D_^22^=+3.7° (*c* 0.30, MeOH); ^1^H-NMR (400 MHz, CD_3_OD, Supplementary Figure S2A): δ (ppm) 7.59 (1H, d, *J*=15.9 Hz, H-7′), 7.04 (1H, d, *J*=2.0 Hz, H-2′), 6.94 (1H, dd, *J*=8.2, 2.0 Hz, H-6′), 6.77 (1H, d, *J*=8.2 Hz, H-5′), 6.31(1H, d, *J*=15.9 Hz, H-8′), 5.36 (1H, brd, *J*=3.6 Hz, H-3), 4.16 (1H, ddd, *J*=9.4, 9.4, 3.5 Hz, H-5), 3.65 (1H, dd, *J*=8.5, 3.3 Hz, H-4), 2.23–2.12 (3H, m, H-2, H-6a), 1.99–1.93 (1H, m, H-6b); ^13^C-NMR (100 MHz, CD_3_OD, Supplementary Figure S2B): δ (ppm) 178.3 (-COOH), 169.0 (C-9′), 149.4 (C-4′), 146.8 (C-7′), 146.7 (C-3′), 127.9 (C-1′), 122.9 (C-6′), 116.4 (C-5′), 115.8 (C-8′), 115.1 (C-2′), 75.4 (C-1), 74.8 (C-4), 73.0 (C-3), 68.3 (C-5), 41.5 (C-6), 36.7 (C-2). The HSQC, HMBC, and COSY spectra are presented in Supplementary Figure S2C, D, and E, respectively.

The spectral properties of 5-*O*-caffeoylquinic acid (5-CafQA, **2**) were as follows: HR-ESI-TOF-MS (Supplementary Figure S1B) (positive) *m*/*z* 355.1021 [M+H]^+^ (calcd. for C_16_H_19_O_9_^+^, 355.1024); UV (MeOH) λ_max_ (logε) 219 nm (4.15), 245 nm (3.98), 330 nm (4.22); [α]_D_^22^=−30.8° (*c* 0.30, MeOH); ^1^H-NMR (400 MHz, CD_3_OD, Supplementary Figure S3A): δ (ppm) 7.56 (1H, d, *J*=15.9 Hz, H-7′), 7.05 (1H, d, *J*=2.0 Hz, H-2′), 6.95 (1H, dd, *J*=8.2, 2.0 Hz, H-6′), 6.78 (1H, d, *J*=8.2 Hz, H-5′), 6.26 (1H, d, *J*=15.9 Hz, H-8′), 5.33 (1H, ddd, *J*=8.9, 8.9, 4.0 Hz, H-5), 4.17 (1H, brs, H-3), 3.73 (1H, dd, *J*=8.3, 2.6 Hz, H-4), 2.24–2.17 (2H, m, H-2a, H-6a), 2.11–2.03 (2H, m, H-2b, H-6b); ^13^C-NMR (100 MHz, CD_3_OD, Supplementary Figure S3B): δ (ppm) 177.0 (-COOH), 168.6 (C-9′), 149.5 (C-4′), 147.1 (C-7′), 146.8 (C-3′), 127.7 (C-1′), 123.0 (C-6′), 116.4 (C-5′), 115.2 (C-8′), 115.1 (C-2′), 76.1 (C-1), 73.4 (C-4), 71.9 (C-5), 71.2 (C-3), 38.7 (C-6), 38.2 (C-2). The HSQC, HMBC, and COSY spectra are presented in Supplementary Figure S3C, D, and E, respectively.

The authentic compounds of 3-CafQA (**1**) and 5-CafQA (**2**) were purchased from Tokyo Chemical Industry Co., Ltd. (Tokyo, Japan), and their NMR spectra and specific optical rotation values ([α]_D_^22^=+5.4° for 3-CafQA (**1**) and −34.7° for 5-CafQA (**2**), *c* 0.30, MeOH) were used as references for the structural confirmation of compounds **1** and **2**.

### Assessment of culture conditions for production of 3-CafQA (**1**) and 5-CafQA (**2**)

To examine the effects of the basal medium, subcultured Pn suspension cells were transferred to the following fresh media with an initial cell density of 5% SCV: MS, B5, and White media. The cells were cultured under the light conditions as described in the “Plant materials and culture conditions” section.

To examine the effects of light conditions, subcultured Pn suspension cells were transferred to fresh B5 medium with an initial cell density of 5% SCV and then cultured for 15 days under the same conditions as described in the “Plant materials and culture conditions” section except for the light conditions. The light conditions were changed depending on the experiments as follows: to examine the effects of light quality, white, red, and blue lights; for the effects of light intensity, 20 (25%, relative intensity compared with the maximum intensity), 40 (50%), and 80 (100%) µmol m^−2^ s^−1^; and for the effects of LD cycle, 0 h/24 h, 8 h/16 h, 16 h/8 h, and 24 h/0 h LD cycles.

To examine the effects of initial cell density, subcultured Pn suspension cells were transferred to fresh B5 medium with initial cell densities of 5%, 10%, and 20% SCVs, and then cultured under the light conditions as described in the “Plant materials and culture conditions” section.

To examine the effects of concentrations of sucrose and macronutrients in B5 medium, subcultured Pn suspension cells were transferred to fresh modified B5 medium with an initial cell density of 20% SCV, and then cultured under the light conditions as described in the “Plant materials and culture conditions” section. Sucrose concentrations were varied from ×1/8 strength (2.5 g l^−1^) to ×8 strength (160 g l^−1^) (×1/8, ×1/4, ×1/2, ×1, ×2, ×4, and ×8). Concentrations of macronutrients in stock I solution for B5 medium (Supplementary Table S1) were varied from ×1/8 strength to ×8 strength (×1/8, ×1/4, ×1/2, ×1, ×2, ×4, and ×8), and those in stock II solution (Supplementary Table S1) were varied in the same range (from ×1/8 to ×8) in the presence of ×1/4 strength stock I.

In every experiment, the cells were collected every 5 days until 30 days, and the contents of 3-CafQA (**1**) and 5-CafQA (**2**) were measured as described in the “Extraction and HPLC analysis” section.

## Results

### Metabolite analysis of Pn suspension cells cultured under light

**Figure 1:**
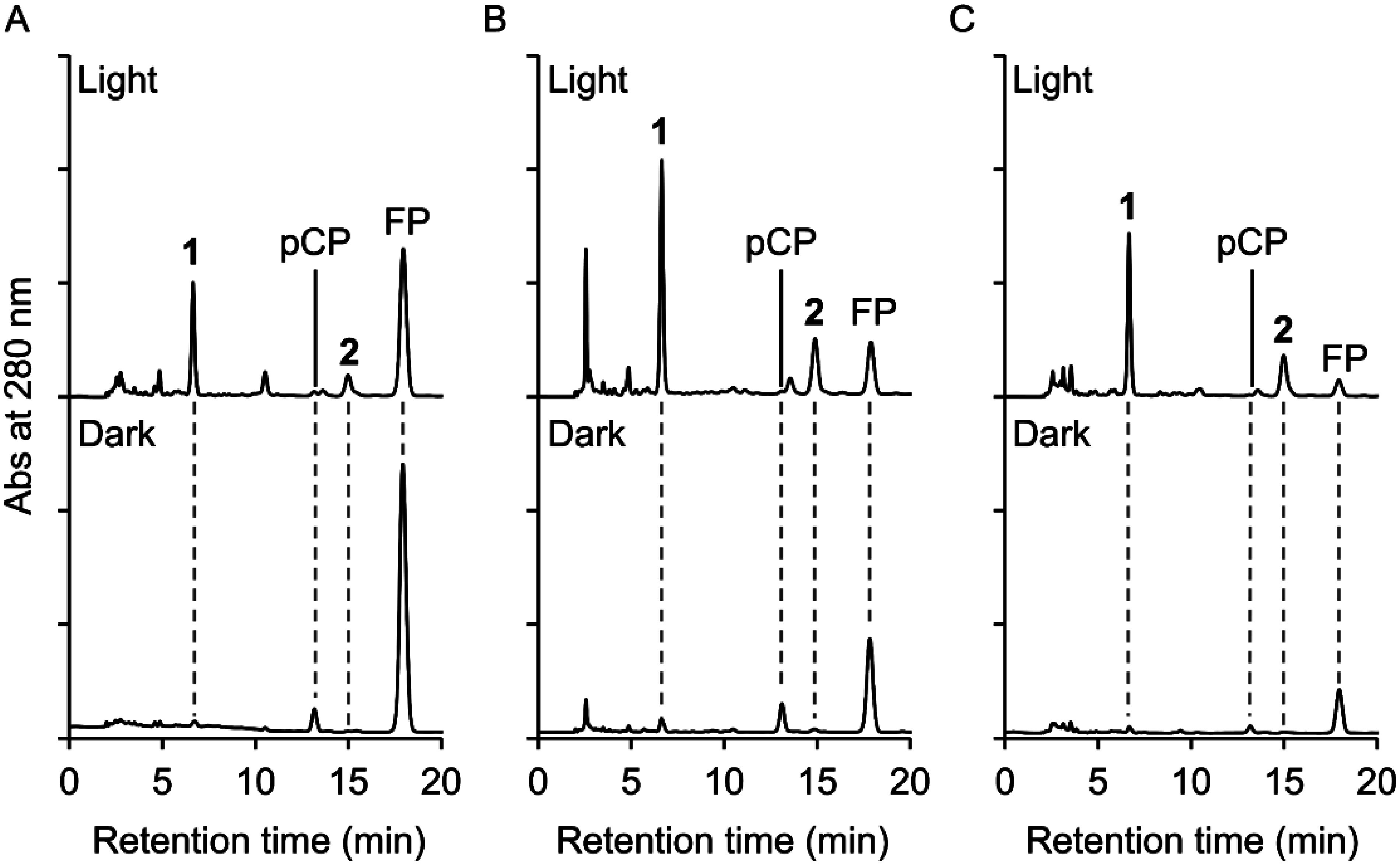
Figure 1. Analysis of the metabolites in the extracts of Pn suspension cells cultured under light or dark conditions. HPLC chromatograms for the extracts from Pn suspension cells cultured in MS (A), B5 (B), and White (C) media for 15 days are presented.

To examine whether light treatment altered the metabolic properties of Pn suspension cells, we first cultured the cells under light and dark conditions in three different basal media (MS, B5, and White media), and the methanolic extracts of the cells were analyzed by reversed-phase HPLC (Figure 1). Under dark conditions, the Pn suspension cells cultured in the three media mainly accumulated feruloylputrescine (FP) and *p*-coumaroylputrescine (pCP), the occurrence of which has been reported previously (Nomura et al. 2013). Under light conditions, in contrast, Pn suspension cells cultured in the three media accumulated substantial levels of compounds **1** and **2**, and the levels of FP and pCP decreased. These results indicated that light treatment altered the metabolic properties of Pn suspension cells. The production levels of compounds **1** and **2** were highest in B5 medium among the three media.

### Isolation and structural elucidation of compounds **1** and **2**

**Figure 2:**
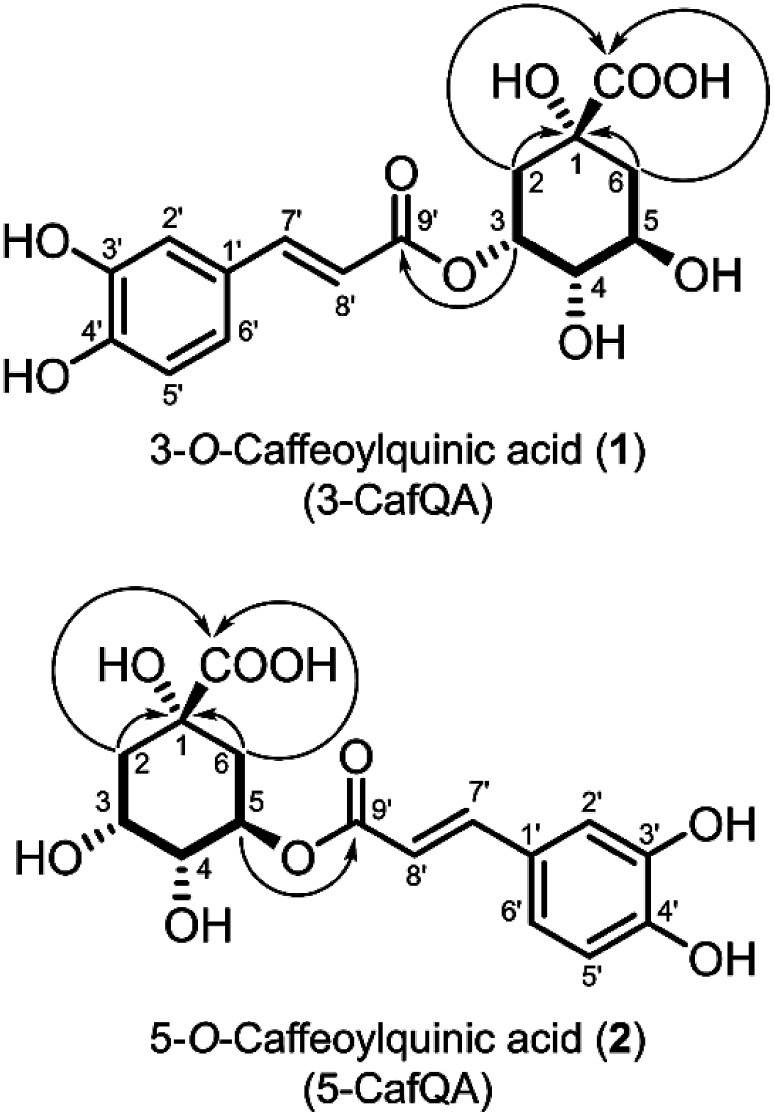
Figure 2. Chemical structures of 3-*O*-caffeoylquinic acid (3-CafQA, **1**) and 5-*O*-caffeoylquinic acid (5-CafQA, **2**). Key COSY and HMBC correlations for quinic acid moiety are indicated by bold lines and arrows (^1^H→^13^C), respectively. Numbering of the quinic acid moiety in this study follows the IUPAC system (Abrankó and Clifford 2017; Clifford et al. 2017). We strongly state this to avoid misunderstanding of the chemical structures reported here in the situation that studies using non-IUPAC numbering are still published.

We purified compounds **1** (108 mg) and **2** (21.0 mg) from 15-day-old Pn suspension cells (95 g FW) cultured under light in B5 medium. The molecular formula of compound **1** was C_16_H_18_O_9_ as determined by HR-ESI-TOF-MS (*m*/*z* 355.1022 [M+H]^+^, Supplementary Figure S1A). The mass spectrum of compound **1** exhibited a diagnostic fragment ion (Onkokesung et al. 2012) at *m*/*z* 163.0399, indicating that compound **1** has a caffeoyl moiety, which was confirmed by the NMR spectra of compound **1**. The ^1^H-NMR spectrum (Supplementary Figure S2A) showed that compound **1** has a 1,3,4-trisubstituted benzene ring and two doublet signals at 7.59 (H-7′) and 6.31 (H-8′) ppm with 15.9 Hz of coupling constant indicated the presence of a *trans* olefin. Both olefin protons (H-7′ and H-8′) showed cross-peaks with the carbonyl carbon (C-9′) at 169.0 ppm and C-1′ at 127.9 ppm in the HMBC spectrum (Supplementary Figure S2D), indicating the presence of a *trans*-caffeoyl moiety in the structure. The ^1^H-NMR and HSQC spectra (Supplementary Figures S2A, C, S4A) indicated the presence of three methine groups (H-3, H-4, and H-5), two methylene groups consisting of non-equivalent geminal protons (H-2 and H-6), and one quaternary carbon (C-1). The COSY spectrum (Supplementary Figure S2E) displayed linkages between C-2, C-3, C-4, C-5, and C-6. According to the HMBC spectrum (Supplementary Figures S2D, S4B), two methylene signals (H-2 and H-6) showed cross peaks with the quaternary carbon signal (C-1) and the carbonyl carbon signal (-COOH), indicating that the two methylene groups and the carbonyl carbon are connected to the quaternary carbon. Therefore, we concluded that the remainder of the structure is quinic acid. The stable chair conformation of quinic acid positions the three methine protons (H-3, H-4, and H-5) as equatorial, axial, and axial, respectively (Abrankó and Clifford 2017; Clifford et al. 2017). They could be distinguished by the coupling width detected in the ^1^H-NMR spectrum; the smallest and largest coupling widths were for H-3 and H-5 (Ortiz et al. 2017). In the ^1^H-NMR spectrum, the signal of the H-3 methine proton with the smallest coupling width was detected in a lower field (5.36 ppm) than that of the H-5 methine proton with the largest coupling width detected at 4.16 ppm and the H-4 methine proton at 3.65 ppm, indicating that an ester bond exists at the C-3 position of quinic acid. This was confirmed by the HMBC correlation between the H-3 (5.36 ppm) of the quinic acid moiety and the C-9′ carbonyl carbon (169.0 ppm) of the caffeoyl moiety. Therefore, we concluded that compound **1** is 3-*O*-caffeoylquinic acid (3-CafQA, Figure 2), which is also known as neochlorogenic acid. The relative and absolute configurations of 3-CafQA (**1**) were confirmed based on the agreement of the NMR spectra and the specific optical rotation value of 3-CafQA (**1**) with those of the authentic compound. The spectral data also accorded well with data reported in the literature (Liu et al. 2020)

Compound **2** had the same molecular formula (C_16_H_18_O_9_) with compound **1**, as determined by HR-ESI-TOF-MS (*m*/*z* 355.1021 [M+H]^+^, Supplementary Figure S1B), indicating that compound **2** is an isomer of compound **1**. The ^1^H- and ^13^C-NMR spectra of compound **2** were similar to those of compound **1**; however, the ^1^H-NMR spectrum of compound **2** differed from that of compound **1**: (1) a downfield shift of a methine proton (H-5) at 4.16 ppm in compound **1** to 5.33 ppm in compound **2**, and (2) an upfield shift of a methine proton (H-3) at 5.36 ppm in compound **1** to 4.17 ppm in compound **2**, indicating that the caffeoyl moiety was ester-linked with a hydroxy group at the C-5 position. Correlations observed in 2D NMR spectra (Supplementary Figures S3C, D, E, S5) supported this structure. Therefore, we concluded that compound **2** is 5-*O*-caffeoylquinic acid (5-CafQA, Figure 2), which is also known as chlorogenic acid. The relative and absolute configurations of 5-CafQA (**2**) were confirmed based on the agreement of the NMR spectra and the specific optical rotation value of 5-CafQA (**2**) with those of the authentic compound. The spectral data also accorded well with data reported in the literature (Liu et al. 2020).

### Effects of light conditions on production of 3-CafQA (**1**) and 5-CafQA (**2**)

**Figure 3:**
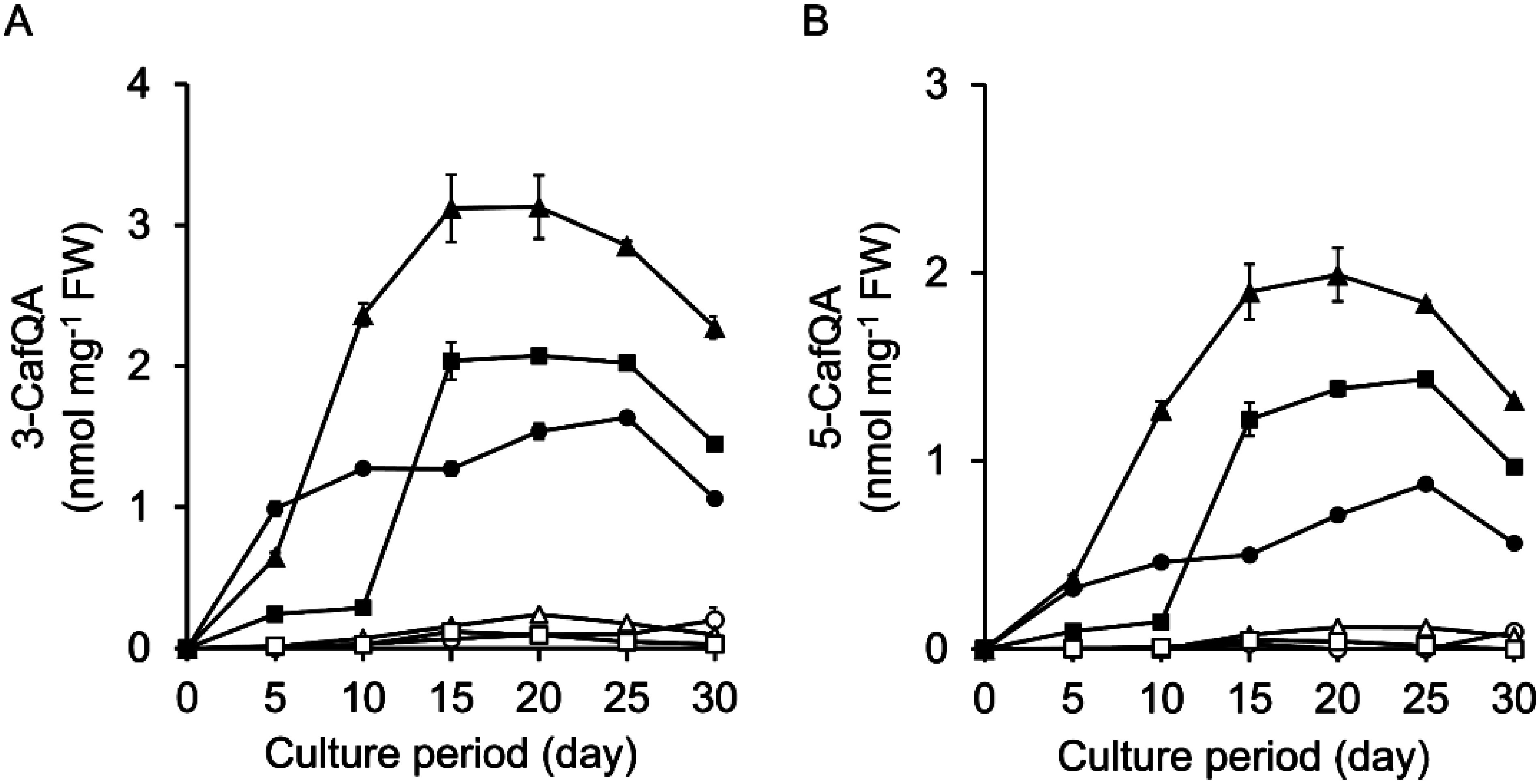
Figure 3. Effects of basal medium and light conditions on the productivity of 3-CafQA (**1**) and 5-CafQA (**2**) in Pn suspension cells. Contents of 3-CafQA (**1**) (A) and 5-CafQA (**2**) (B) in the suspension cells cultured in B5 (triangles), MS (circles), and White (squares) media under light (filled symbols) and dark (open symbols) conditions are shown. Initial cell density was set to 5% SCV. Data are presented as the mean±SD (*n*=3).

Pn suspension cells were cultured for 30 days in B5, MS, and White media under light or in the dark. Cell proliferation was severely suppressed under light (Supplementary Figure S6). However, 3-CafQA (**1**) and 5-CafQA (**2**) were substantially accumulated in the cells cultured under light in all of the three basal media, whereas the compounds were detected at low levels or not detected in cells cultured in the dark (Figure 3). The production of both compounds under light was greater in the cells cultured in B5 medium than in MS and White media. The 3-CafQA (**1**) and 5-CafQA (**2**) contents increased after initiation of the culture under light and peaked at approximately days 15–25. The maximum contents of 3-CafQA (**1**) and 5-CafQA (**2**) were 3.1 nmol mg^−1^ FW and 2.0 nmol mg^−1^ FW, respectively, at day 20 under light in B5 medium. The contents of FP and pCP, major compounds in Pn cells cultured in the dark, were also measured. Induction of FP and pCP biosynthesis was not observed under light and instead was suppressed relative to cells cultured in the dark (Supplementary Figure S7).

**Figure 4:**
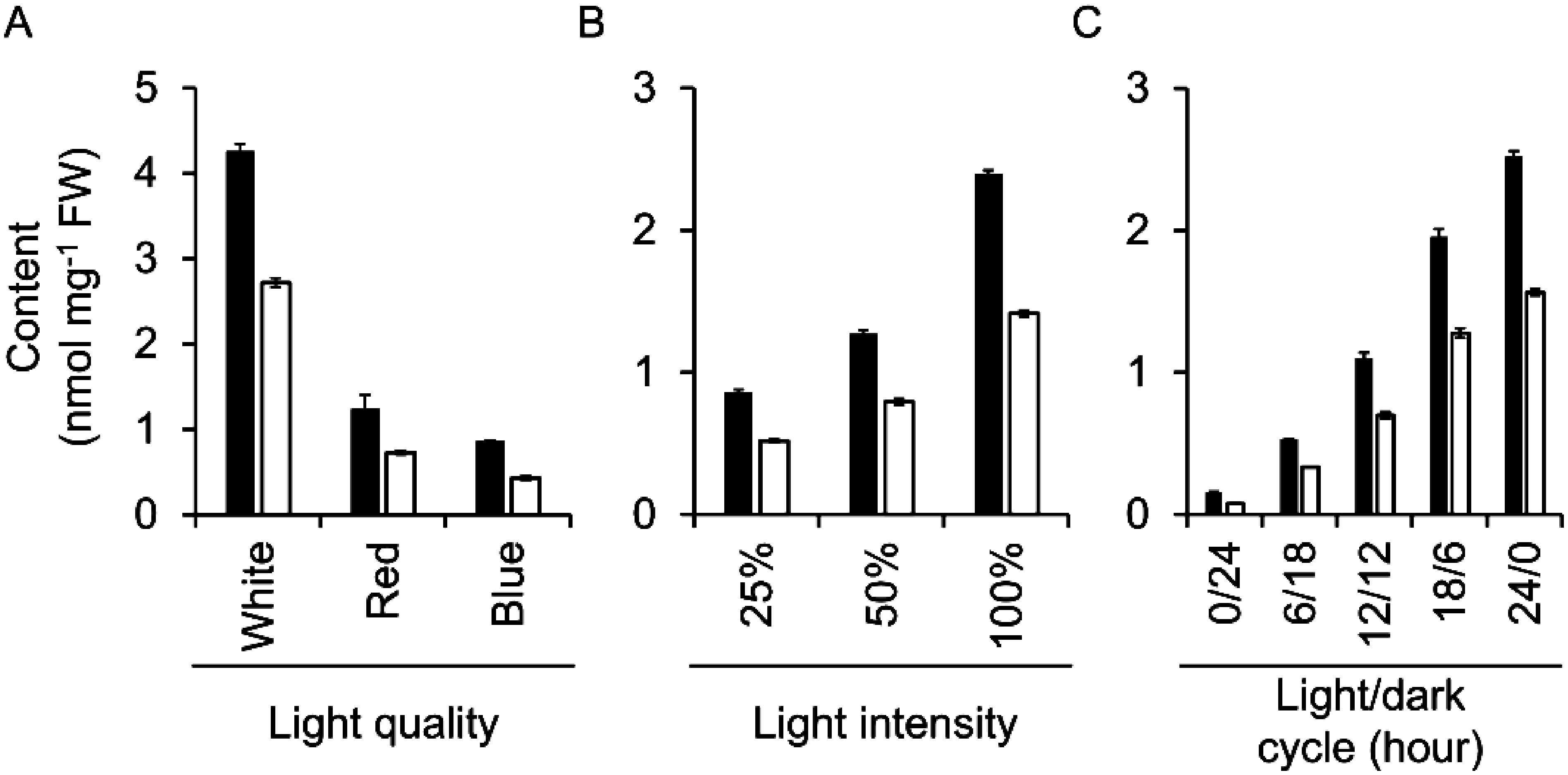
Figure 4. Effects of light conditions on the productivity of 3-CafQA (**1**) and 5-CafQA (**2**) in Pn suspension cells. Contents of 3-CafQA (**1**) (filled bars) and 5-CafQA (**2**) (open bars) in the Pn suspension cells cultured in B5 medium for 15 days under various light conditions are shown: light quality (A), light intensity (B), and light/dark cycle (C). Initial cell density was set to 5% SCV. Data are presented as the mean±SD (*n*=3).

We next examined the effects of light conditions (i.e., light quality, intensity, and LD cycle) on the production of 3-CafQA (**1**) and 5-CafQA (**2**). Initially, Pn suspension cells were cultured for 15 days in B5 medium under various light-source colors. Both 3-CafQA (**1**) and 5-CafQA (**2**) were produced under white, red, and blue light, but the contents of both compounds were higher in the cells cultured under white light than those under red or blue light (Figure 4A), indicating that white light is most suitable for the production of 3-CafQA (**1**) and 5-CafQA (**2**). White light intensity and LD cycle also affected the production of 3-CafQA (**1**) and 5-CafQA (**2**). The contents of both compounds increased together with the increase in the light intensity (Figure 4B) and the light period (Figure 4C). Similarly, the production titers of 3-CafQA (**1**) and 5-CafQA (**2**) increased under the conditions showing the higher contents of both compounds (Supplementary Figure S8).

### Effects of medium components on production of 3-CafQA (**1**) and 5-CafQA (**2**)

**Figure 5:**
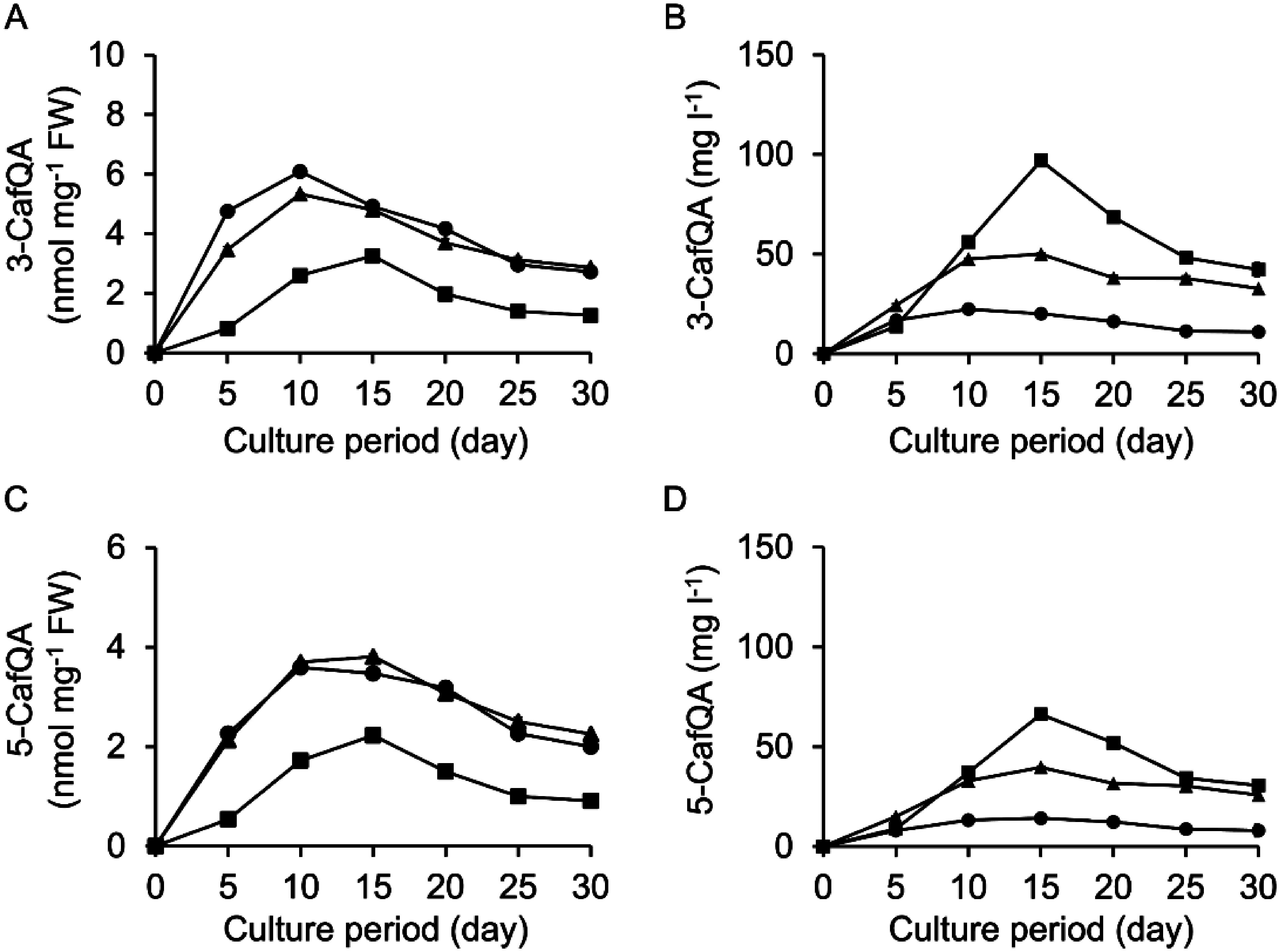
Figure 5. Effects of initial cell density on the productivity of 3-CafQA (**1**) and 5-CafQA (**2**) in Pn suspension cells. Contents of 3-CafQA (**1**) and 5-CafQA (**2**) (A, C) and production titers of 3-CafQA (**1**) and 5-CafQA (**2**) (B, D) in suspension cells cultured in B5 medium under light with different initial cell densities are shown. Initial cell densities were set to 5% (circles), 10% (triangles), and 20% (squares) SCVs. Data are presented as the mean±SD (*n*=3).

The dependency of the production levels of 3-CafQA (**1**) and 5-CafQA (**2**) on culture conditions other than the light conditions was examined. The light conditions used followed the results described in the preceding section (continuous white light with 100% intensity). Initially, the effect of initial cell density on the contents and production titers of 3-CafQA (**1**) and 5-CafQA (**2**) was examined with initial cell densities set to 5%, 10%, and 20% SCVs. The contents of 3-CafQA (**1**) and 5-CafQA (**2**) decreased under the 20% SCV conditions compared with those under the 5% and 10% SCV conditions (Figure 5A, C). In contrast, the maximum production titers of 3-CafQA (**1**) and 5-CafQA (**2**) in the cells peaked at 96.9 and 66.3 mg l^−1^, respectively, at day 15 under the 20% SCV conditions (Figure 5B, D), because the cell amount under the 20% SCV conditions was higher than those under the 5% and 10% SCV conditions. Thus, in the following experiments, the initial cell density was set to 20% SCV.

**Figure 6:**
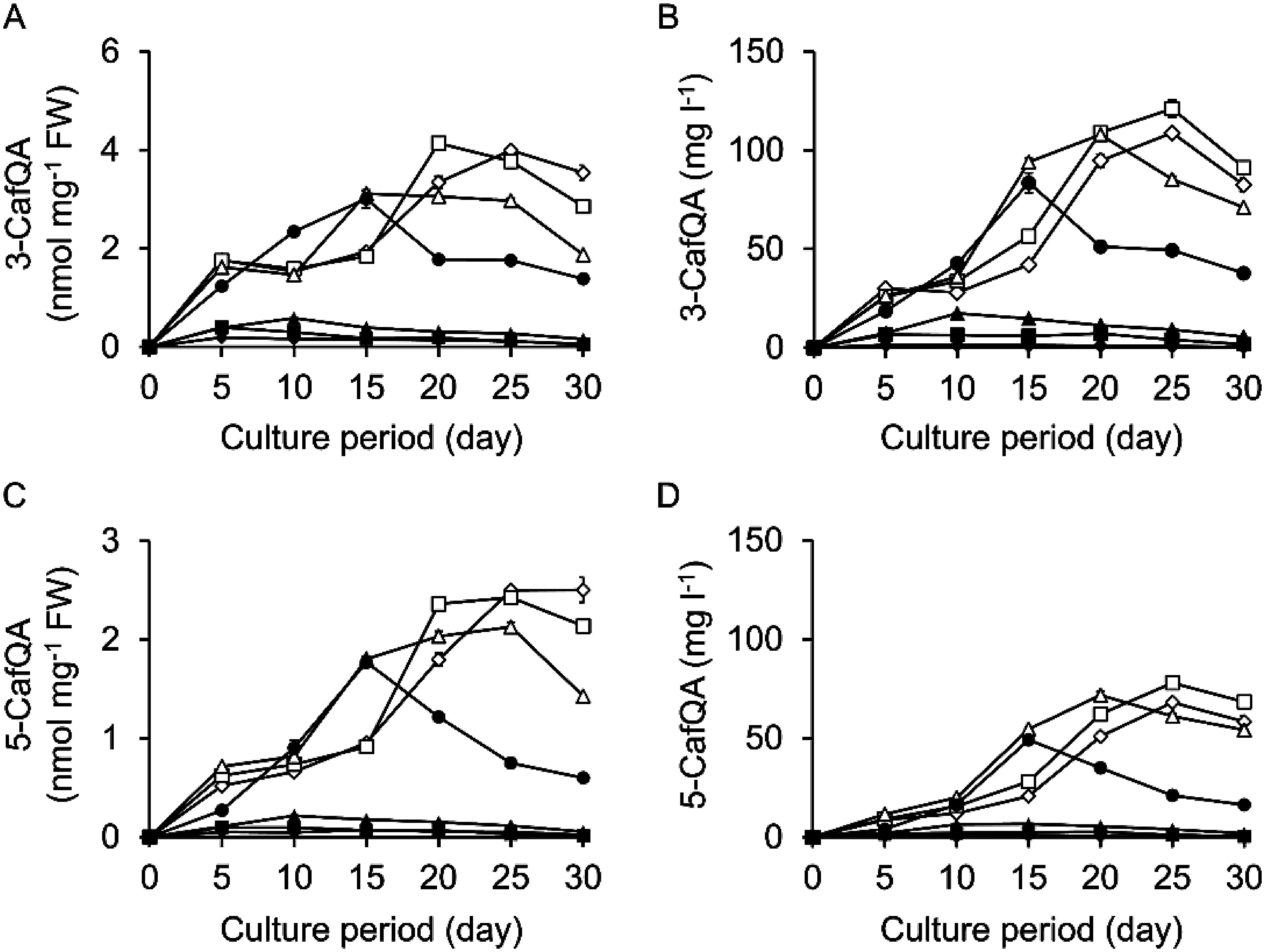
Figure 6. Effects of stock I strength in B5 medium on the productivity of 3-CafQA (**1**) and 5-CafQA (**2**) in Pn suspension cells. Contents of 3-CafQA (**1**) and 5-CafQA (**2**) (A, C) and production titers of 3-CafQA (**1**) and 5-CafQA (**2**) (B, D) in suspension cells cultured under light in B5 medium with different stock I strengths are shown. Stock I strengths were as follows: ×1/8 (open diamonds), ×1/4 (open squares), ×1/2 (open triangles), ×1 (filled circles), ×2 (filled triangles), ×4 (filled squares), ×8 (filled diamonds). Initial cell density was set to 20% SCV. Data are presented as the mean±SD (*n*=3).

Next, we examined the effects of concentrations of sucrose and macronutrients in B5 medium on the contents and production titers of 3-CafQA (**1**) and 5-CafQA (**2**). The macronutrients in B5 medium are KNO_3_, (NH_4_)_2_SO_4_, MgSO_4_, and NaH_2_PO_4_, which are contained in the stock I solution, and CaCl_2_, which is contained in the stock II solution. The time-course changes in the contents and production titers of 3-CafQA (**1**) and 5-CafQA (**2**) were examined in Pn suspension cells cultured in B5 medium. Under different sucrose concentrations (ranging from ×1/8 to ×8 strength), the contents and production titers of 3-CafQA (**1**) and 5-CafQA (**2**) were almost comparable under the ×1 and ×2 strength sucrose conditions and decreased under the other conditions (Supplementary Figure S9). Thus, we adopted ×1 strength sucrose conditions in the assessment of macronutrient concentrations. When the stock I strength was varied (from ×1/8 to ×8 strength), the contents of 3-CafQA (**1**) and 5-CafQA (**2**) increased under low stock I strength conditions, and the maximum production titers reached 121 and 77.9 mg l^−1^, respectively, in cells cultured in medium with ×1/4 strength stock I conditions at day 20 (Figure 6). Therefore, we examined the effects of stock II strength under the ×1/4 strength stock I conditions. When the stock II strength was varied (from ×1/8 to ×8 strength), the contents and production titers of 3-CafQA (**1**) and 5-CafQA (**2**) did not change notably (Supplementary Figure S10).

## Discussion

We previously reported that Pn suspension cells produce the hydroxycinnamoylputrescines, FP and pCP, as major compounds when cultured in the dark (Nomura et al. 2013). In the present study, the biosynthesis of two caffeoylquinic acids, 3-CafQA (**1**) and 5-CafQA (**2**), was strongly activated in Pn suspension cells cultured under light. The maximum production titers of 3-CafQA (**1**) and 5-CafQA (**2**) reached 121 and 77.9 mg l^−1^, respectively, by modifying the culture conditions, including adjustment of the initial cell density and the macronutrient concentration. The compounds 3-CafQA (**1**) and 5-CafQA (**2**) are known as neochlorogenic acid and chlorogenic acid, respectively, and are produced in diverse plant species. As these compounds exhibit various bioactivities (e.g., antioxidative, antimicrobial, and anti-inflammatory activities) (Wang et al. 2022), they have attracted attention as cosmetic and functional food ingredients. The present findings highlight that Pn cells may be an alternative host for the bioproduction of caffeoylquinic acids.

The light-induced accumulation of 3-CafQA (**1**) and 5-CafQA (**2**) is reasonable, on account of their antioxidative activity, to decrease oxidative stress in the Pn cells, because light treatment causes oxidative stress via generation of reactive oxygen species in plants (Foyer et al. 1994). The antioxidative activity of phenylpropanoid-derived compounds is usually affected by the structure of the phenolic moieties; for example, the activity of caffeic acid is higher than those of ferulic and *p*-coumaric acids (Chen et al. 2020). Thus, Pn cells likely adapted to the light conditions by accumulating caffeoyl-CoA-derived compounds, 3-CafQA (**1**) and 5-CafQA (**2**), rather than feruloyl- and *p*-coumaroyl-CoAs-derived compounds, FP and pCP.

Regarding 5-CafQA (**2**) biosynthesis, three pathways have been proposed: (1) condensation of caffeoyl-CoA and quinic acid by hydroxycinnamoyl-CoA:quinate hydroxycinnamoyltransferase (HQT) (Hoffmann et al. 2003; Ulbrich and Zenk 1979), (2) hydroxylation of *p*-coumaroylquinic acid by *p*-coumarate 3′-hydroxylase, and (3) transacylation of 1-*O*-caffeoylglucose ester to quinic acid by hydroxycinnamoyl glucose:quinate hydroxycinnamoyltransferase (Villegas and Kojima 1986). Although the major pathway for 5-CafQA (**2**) biosynthesis is unclear in Pn suspension cells, one of these pathways is likely activated by light treatment. The biosynthetic pathway of 3-CafQA (**1**), a regioisomer of 5-CafQA (**2**), has not been elucidated. Recently, we reported that a HQT enzyme catalyzes the formation of 3-*O*-feruloyl- and 3-*O*-*p*-coumaroylquinic acids from feruloyl- and *p*-coumaroyl-CoAs and quinic acid in a regiospecific manner in the bamboo species *Bambusa multiplex* (Nomura et al. 2022). However, caffeoyl-CoA is inferior to feruoyl- and *p*-coumaroyl-CoAs as a substrate for this enzyme (BmHQT1). Given that neither 3-*O*-feruloyl- nor 3-*O*-*p*-coumaroylquinic acids were detected in the light-treated Pn cells, Pn cells may have a BmHQT1-like enzyme that prefers caffeoyl-CoA as the acyl-donor, and the light-induced expression of the corresponding gene appears to have led to the accumulation of 3-CafQA (**1**) in Pn cells.

Regardless of the acylation positions of quinic acid, only caffeoylquinate esters were detected in response to the light treatment of Pn suspension cells. This result can be explained by the substrate specificities of the ester-forming enzymes. However, it is also likely that the supply of caffeoyl-CoA surpasses that of feruloyl- and *p*-coumaroyl-CoAs, which is supported by the observation that the biosynthetic levels of FP and pCP markedly decreased in Pn cells cultured under light.

The light-induced activation of the biosynthesis of caffeoylquinic acids is a useful trait for the bioproduction of exogenous caffeoyl derivatives in transformed Pn cells based on the rational metabolic-flow switching strategy (Nomura et al. 2018). Utilizing this strategy, we previously succeeded in the bioproduction of exogenous phenylpropanoid-related compounds in transformed Pn cells, but the compounds formed were, without exception, those derived from feruloyl- and *p*-coumaroyl-CoAs (Kitaoka et al. 2020, 2021; Nomura et al. 2018; Ube et al. 2024). This is because the metabolic-flow switching targeted the intermediates for biosynthesis of FP and pCP, major secondary metabolites in Pn suspension cells cultured in the dark. The ability of Pn cells to biosynthesize caffeoylquinic acids suggests that the targeted bioproduction of exogenous compounds with a caffeoyl moiety in transformed Pn cells is feasible. It is also noteworthy that the structure of the hydroxycinnamic acid-derived moiety can be controlled by culturing the cells under light and dark conditions. To target compounds derived from caffeoyl-CoA, the culture conditions optimized for elevated 3-CafQA (**1**) and 5-CafQA (**2**) production, in particular including continuous white light and ×1/4 strength of stock I macronutrients, will allow production of the target compound at high levels. Elucidation of the metabolic pathways that are activated by stress factors other than light and low-strength macronutrients (e.g., heat, cold, and biotic elicitors) will further expand the structural variety of exogenous compounds produced via the rational metabolic-flow switching strategy in Pn cells.

## Abbreviations

BaPAD: *Bacillus amyloliquefaciens* phenolic acid decarboxylase
BmHQT: *Bambusa multiplex* hydroxycinnamoyl-CoA:quinate hydroxycinnamoyltransferase
5-CafQA: 5-*O*-caffeoylquinic acid
FP: feruloylputrescine
FW: fresh weight
HvACT: *Hordeum vulgare* agmatine coumaroyltransferase
ODS: octadecylsilyl
pCP: *p*-coumaroylputrescine
Pn: *Phyllostachys nigra*
PpHCHL: *Pseudomonas putida* hydroxycinnamoyl-CoA hydratase/lyase
SCV: sedimented cell volume
3-CafQA: 3-*O*-caffeoylquinic acid

## Description of Supplementary Files

Supplementary Table S1. Components of MS, B5, and White media.

Supplementary Figure S1. Mass spectra (HR-ESI-TOF-MS) of 3-CafQA (**1**) (A) and 5-CafQA (**2**) (B).

Supplementary Figure S2. NMR spectra of 3-CafQA (**1**).

Supplementary Figure S3. NMR spectra of 5-CafQA (**2**).

Supplementary Figure S4. Magnified HSQC (A) and HMBC (B) spectra of 3-CafQA (**1**).

Supplementary Figure S5. Magnified HSQC (A) and HMBC (B) spectra of 5-CafQA (**2**).

Supplementary Figure S6. Growth profiles of Pn suspension cells cultured under light or dark conditions in different basal media.

Supplementary Figure S7. Time-course of changes in FP and pCP contents in Pn suspension cells cultured under light or dark conditions in different basal media.

Supplementary Figure S8. Effects of light conditions on production titers of 3-CafQA (**1**) and 5-CafQA (**2**) in Pn suspension cells.

Supplementary Figure S9. Effects of sucrose strength on production of 3-CafQA (**1**) and 5-CafQA (**2**) in Pn suspension cells.

Supplementary Figure S10. Effects of stock II strength on production of 3-CafQA (**1**) and 5-CafQA (**2**) in Pn suspension cells.
