# Supplementary material for: Occurrence of caffeoylquinic acids in bamboo suspension cells cultured under light
Source: Plant Biotechnol (Tokyo). 2024 Dec 25;41(4):417–24. doi: 10.5511/plantbiotechnology.24.0812a (PMC11897715; doi:10.5511/plantbiotechnology.24.0812a)
Supplement: Supplementary Data [file plantbiotechnology-41-4-24.0812a-s001.pdf]

The Supplementary File

Occurrence of caffeoylquinic acids in bamboo suspension cells cultured under light

Naoki Ube[1,*], Yasuo Kato[1], Taiji Nomura[1]

[1]Biotechnology Research Center and Department of Biotechnology, Toyama Prefectural University, 5180 Kurokawa, Imizu, Toyama 939-0398, Japan

*Corresponding author. Tel.: +81-766-56-7500 (ext. 1517)

E-mail address: nube@pu-toyama.ac.jp

Supplementary Table S1. Components of MS, B5, and White media

MS		B5		White	
Components	$mg\ l^{-1}$	Components	$mg\ l^{-1}$	Components	$mg\ l^{-1}$
KNO_3	1900	KNO_3 (I)	2500	KNO_3	80
NH_4NO_3	1650	$(NH_4)_2SO_4$ (I)	134	$Ca(NO_3)_2 \cdot 4H_2O$	300
$MgSO_4 \cdot 7H_2O$	370	$MgSO_4 \cdot 7H_2O$ (I)	250	$MgSO_4 \cdot 7H_2O$	720
KH_2PO_4	170	$NaH_2PO_4 \cdot H_2O$ (I)	150	KH_2PO_4	68
$CaCl_2 \cdot 2H_2O$	440	$CaCl_2 \cdot 2H_2O$ (II)	150	$NaH_2PO_4 \cdot H_2O$	16.5
H_3BO_3	6.2	H_3BO_3	3.0	H_3BO_3	1.5
$MnSO_4 \cdot 4H_2O$	22.3	$MnSO_4 \cdot H_2O$	10	$MnSO_4 \cdot H_2O$	5.3
$ZnSO_4 \cdot 7H_2O$	8.6	$ZnSO_4 \cdot 7H_2O$	2.0	$ZnSO_4 \cdot 7H_2O$	3.0
KI	0.83	KI	0.75	KCl	65
$Na_2MoO_4 \cdot 2H_2O$	0.25	$Na_2MoO_4 \cdot 2H_2O$	0.25	$Fe_2(SO_4)_3$	32.5
$CuSO_4 \cdot 5H_2O$	0.025	$CuSO_4 \cdot 5H_2O$	0.025	Na_2SO_4	200
$CoCl_2 \cdot 6H_2O$	0.025	$CoCl_2 \cdot 6H_2O$	0.025	Nicotinic acid	5.0
$FeSO_4 \cdot 7H_2O$	27.8	$FeSO_4 \cdot 7H_2O$	27.8	Pyridoxine・HCl	1.0
Na_2・EDTA	37.3	Na_2・EDTA	37.3	Thiamine・HCl	1.0
Nicotinic acid	0.50	Nicotinic acid	1.0	Pantothenic acid Ca	1.0
Pyridoxine・HCl	0.50	Pyridoxine・HCl	1.0	Glycine	30
Thiamine・HCl	0.10	Thiamine・HCl	10	Sucrose	20000
Glycine	2.0	*myo* -Inositol	100		
myo -Inositol	100	Sucrose	20000		
Sucrose	30000				

"(I)" and "(II)" indicate concentrated stock solutions (stock I and II, respectively) for preparation of B5 medium.

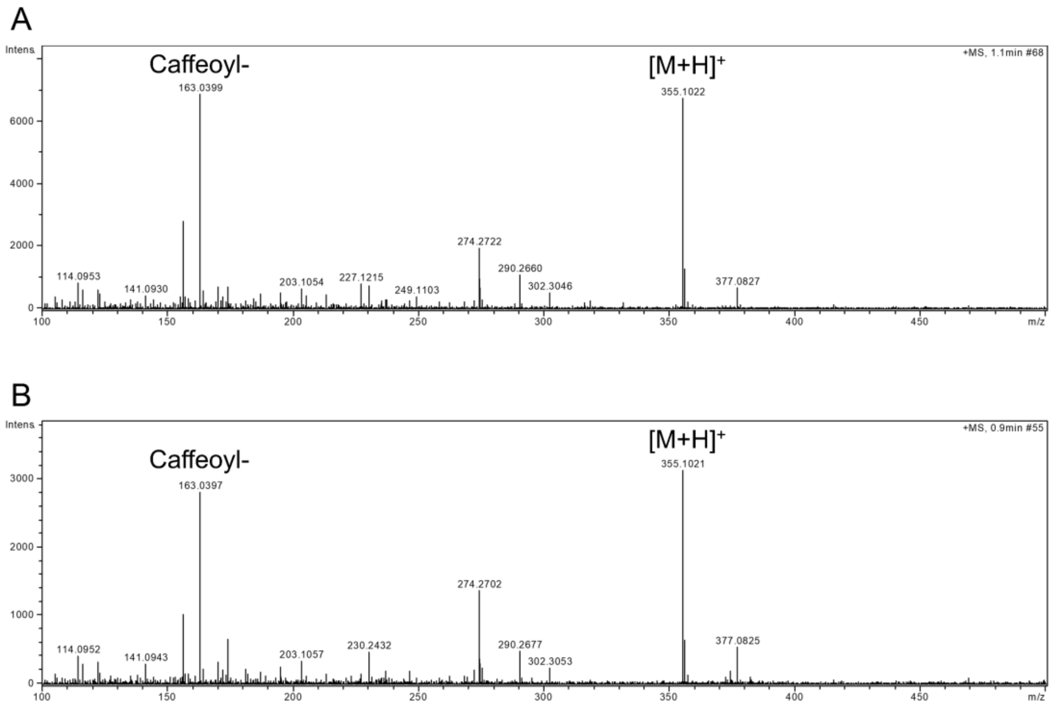

Supplementary Figure S1. Mass spectra (HR-ESI-TOF-MS) of 3-CafQA (1) (A) and 5-CafQA (2) (B).

A

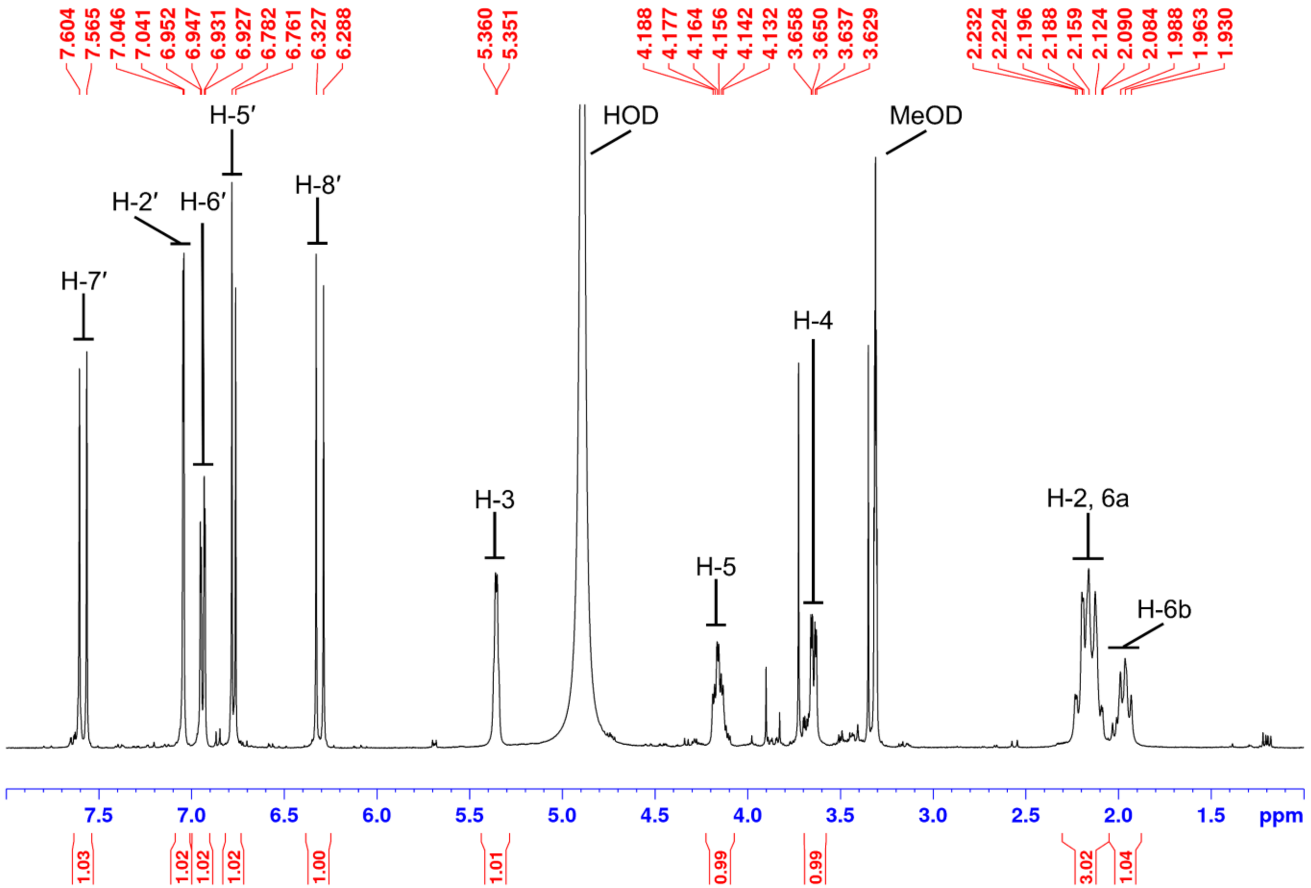

Supplementary Figure S2. NMR spectra of 3-CafQA (1).

(A) ^{1}H-NMR spectrum (400 MHz, CD_3OD)

B

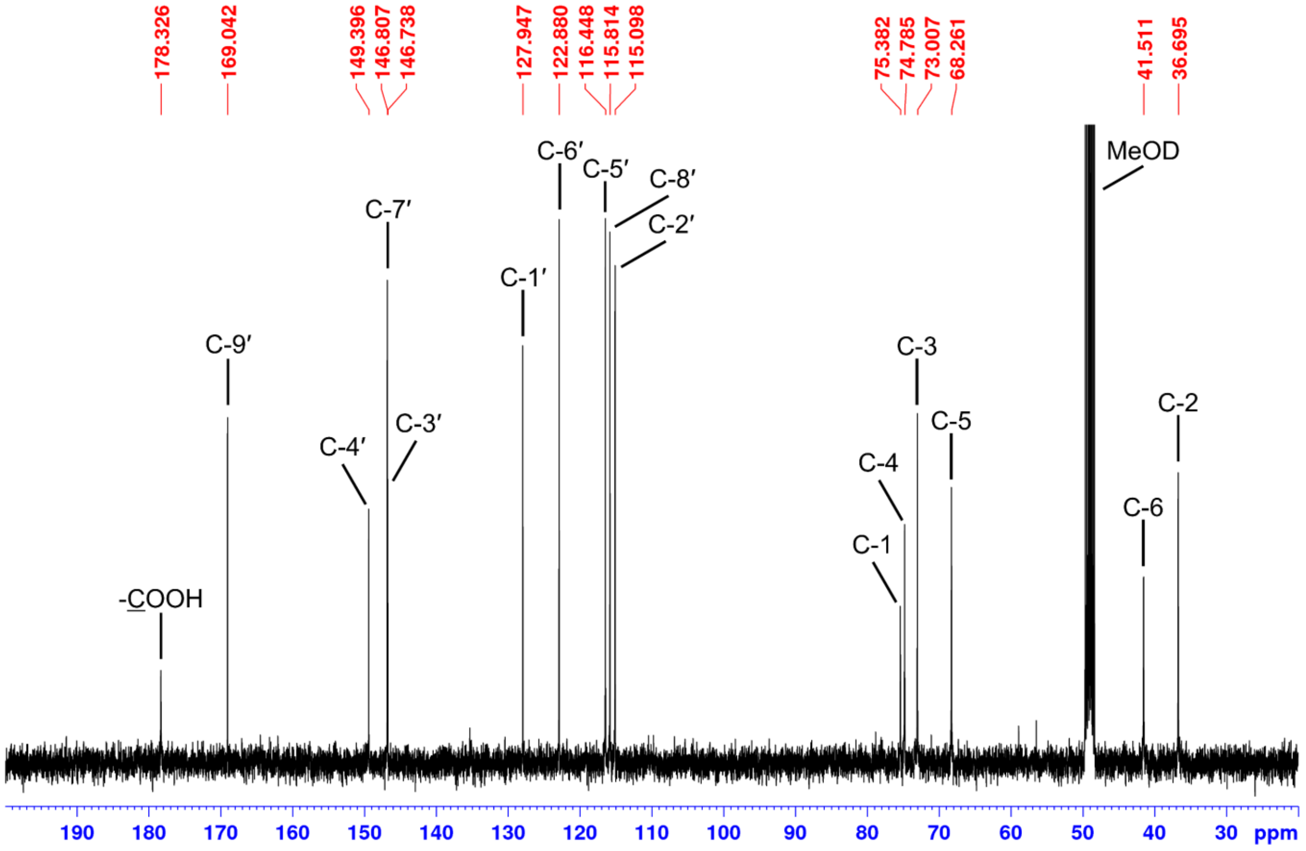

Supplementary Figure S2. (continued) NMR spectra of 3-CafQA (1).

(B) ^{13}C-NMR spectrum (100 MHz, CD_3OD)

C

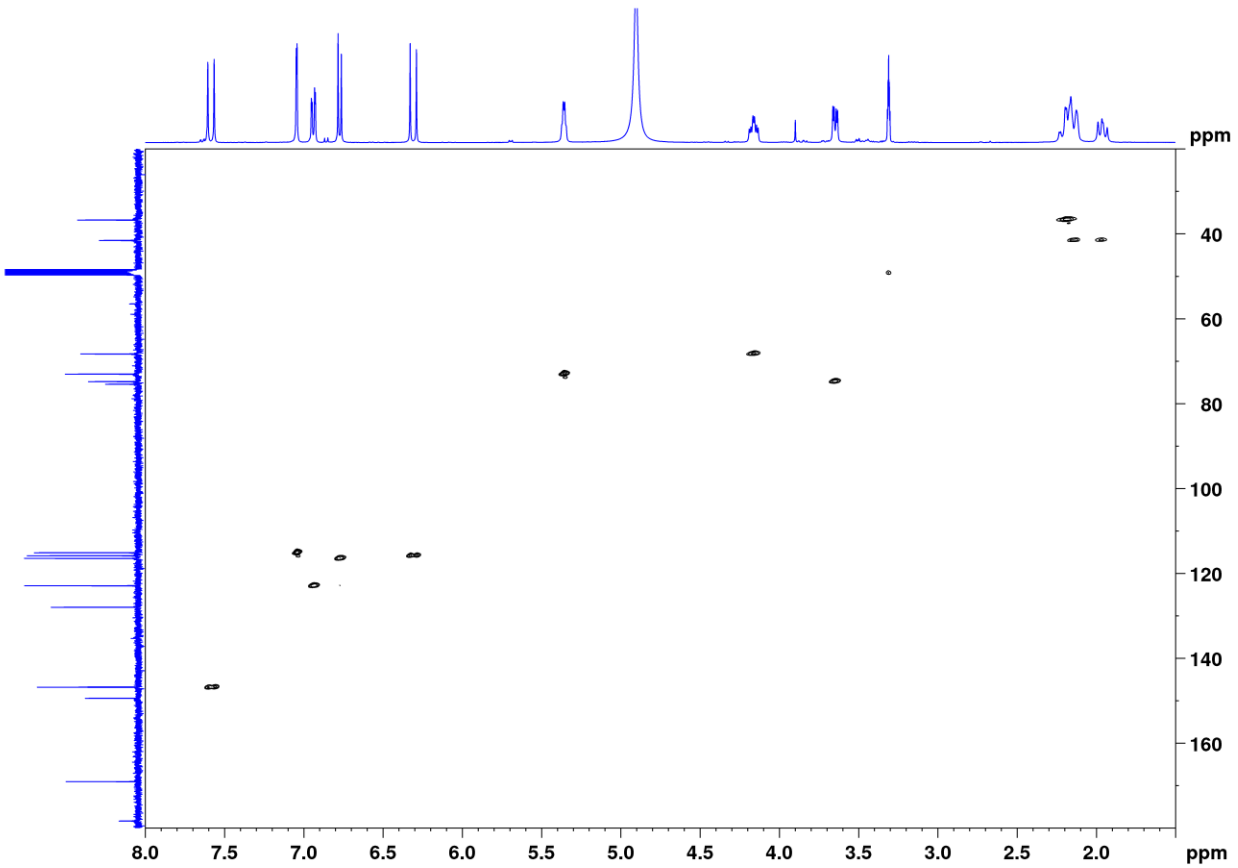

Supplementary Figure S2. (continued) NMR spectra of 3-CafQA (1).

(C) HSQC spectrum

D

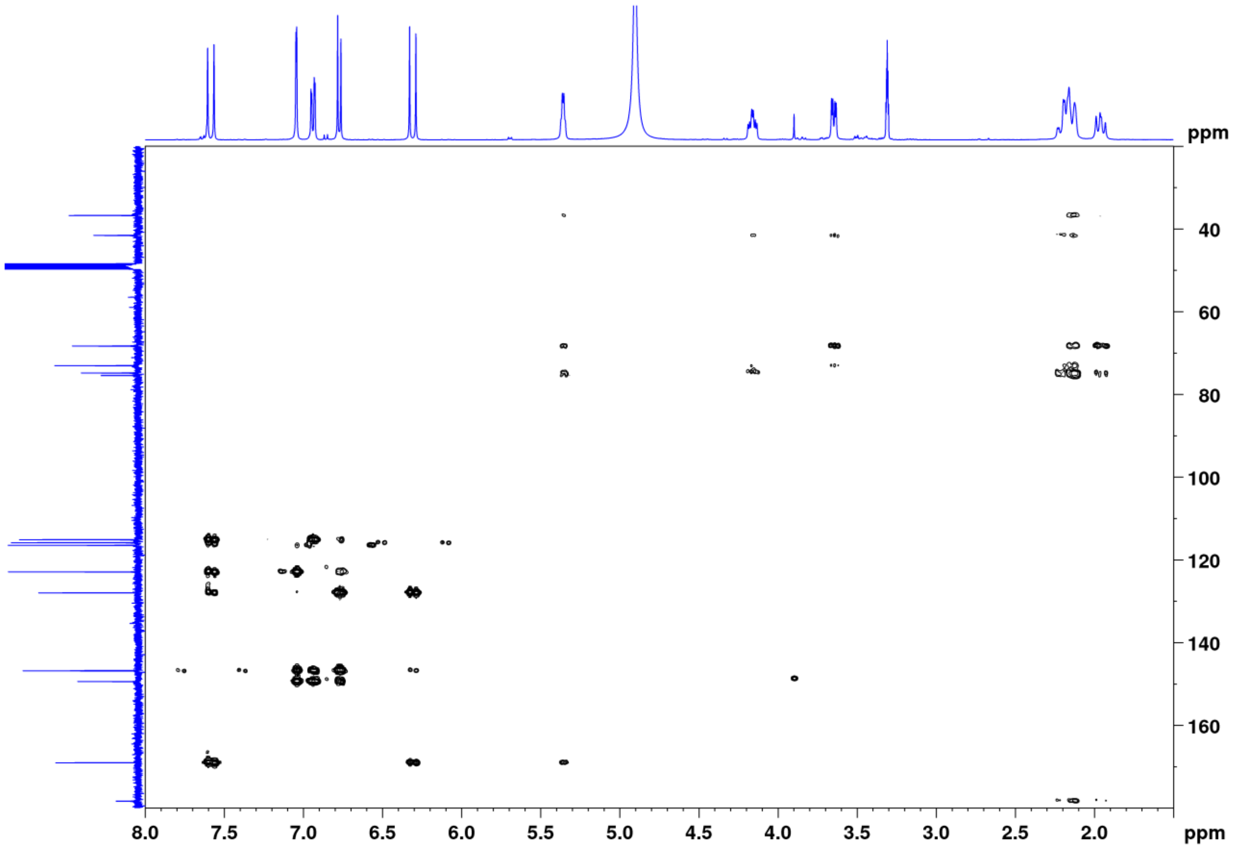

Supplementary Figure S2. (continued) NMR spectra of 3-CafQA (1).

(D) HMBC spectrum

E

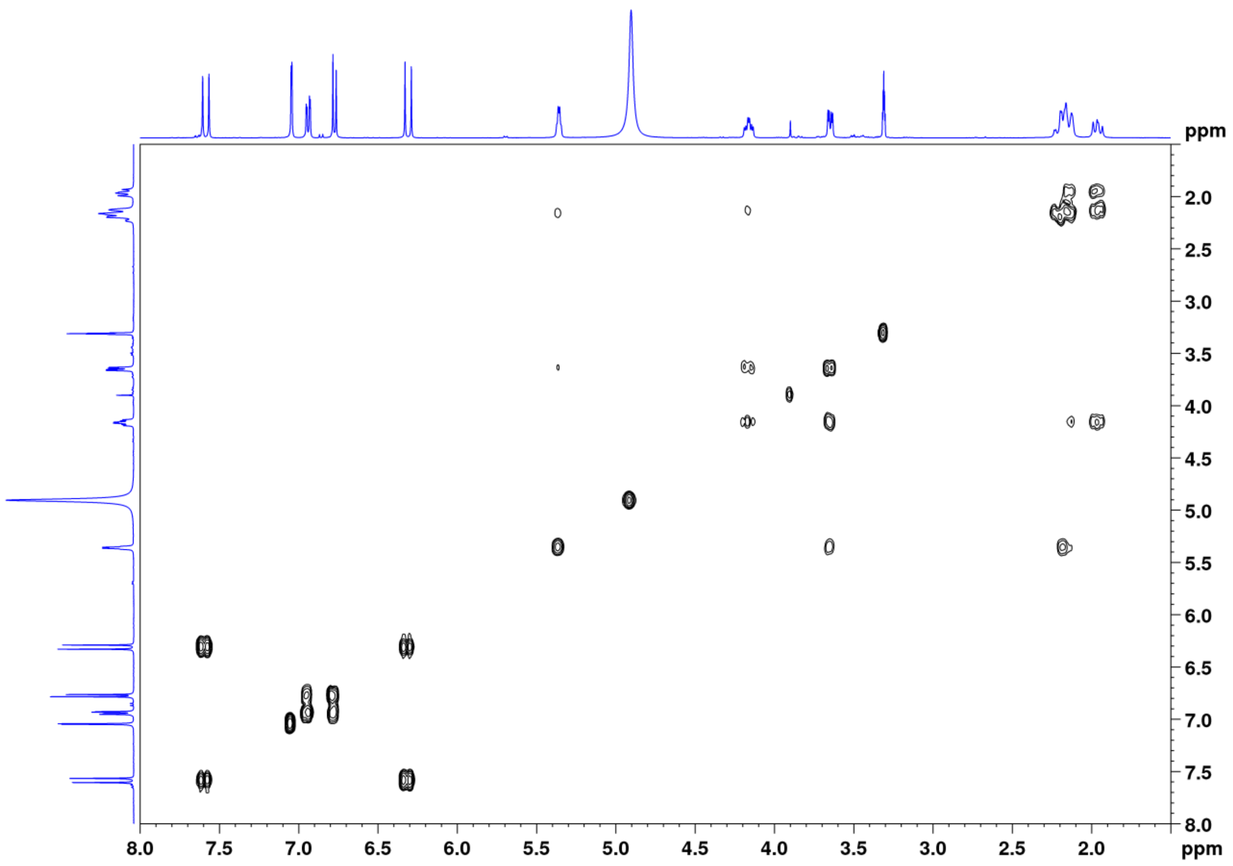

Supplementary Figure S2. (continued) NMR spectra of 3-CafQA (1).

(E) COSY spectrum

A

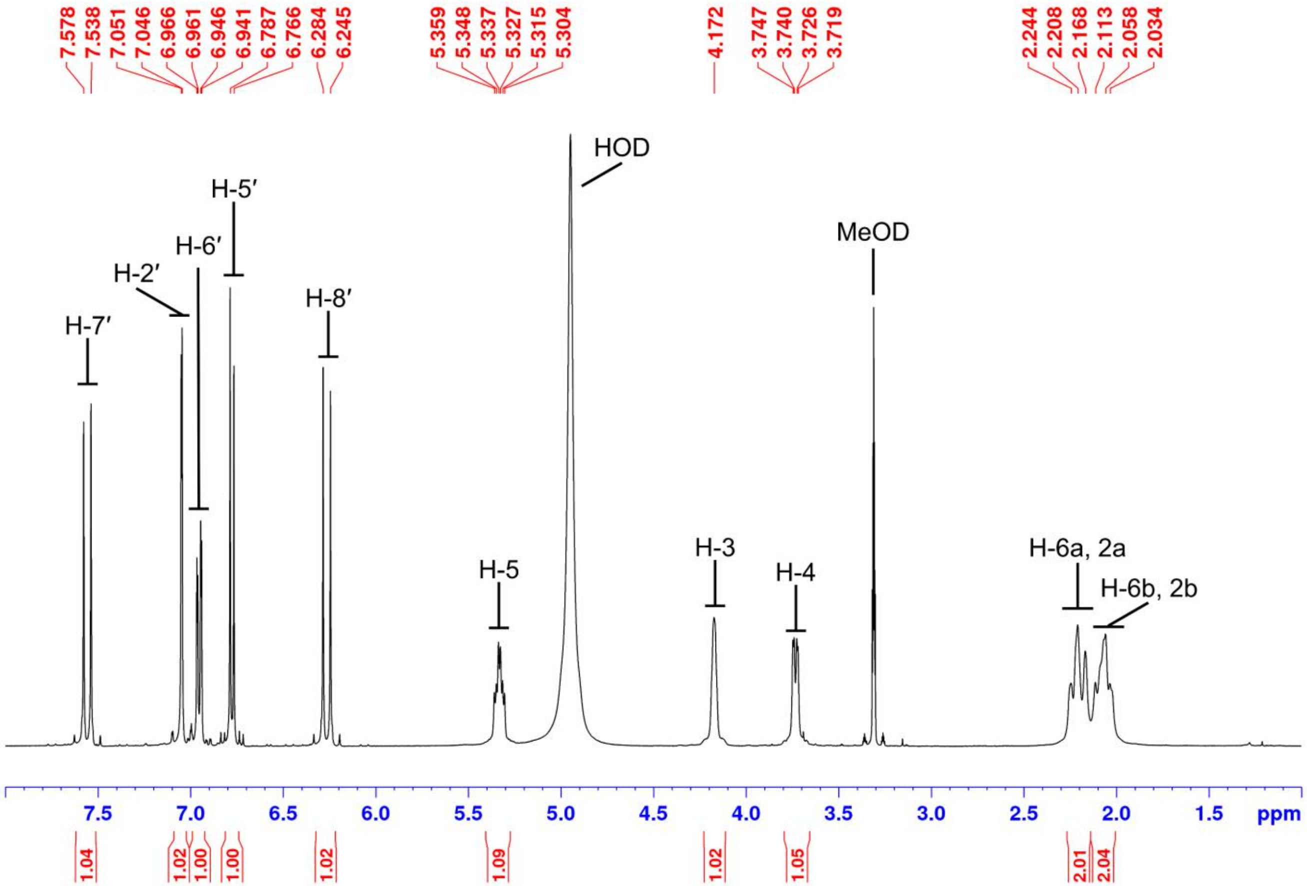

Supplementary Figure S3. NMR spectra of 5-CafQA (2).

(A) ^{1}H-NMR spectrum (400 MHz, CD_3OD)

B

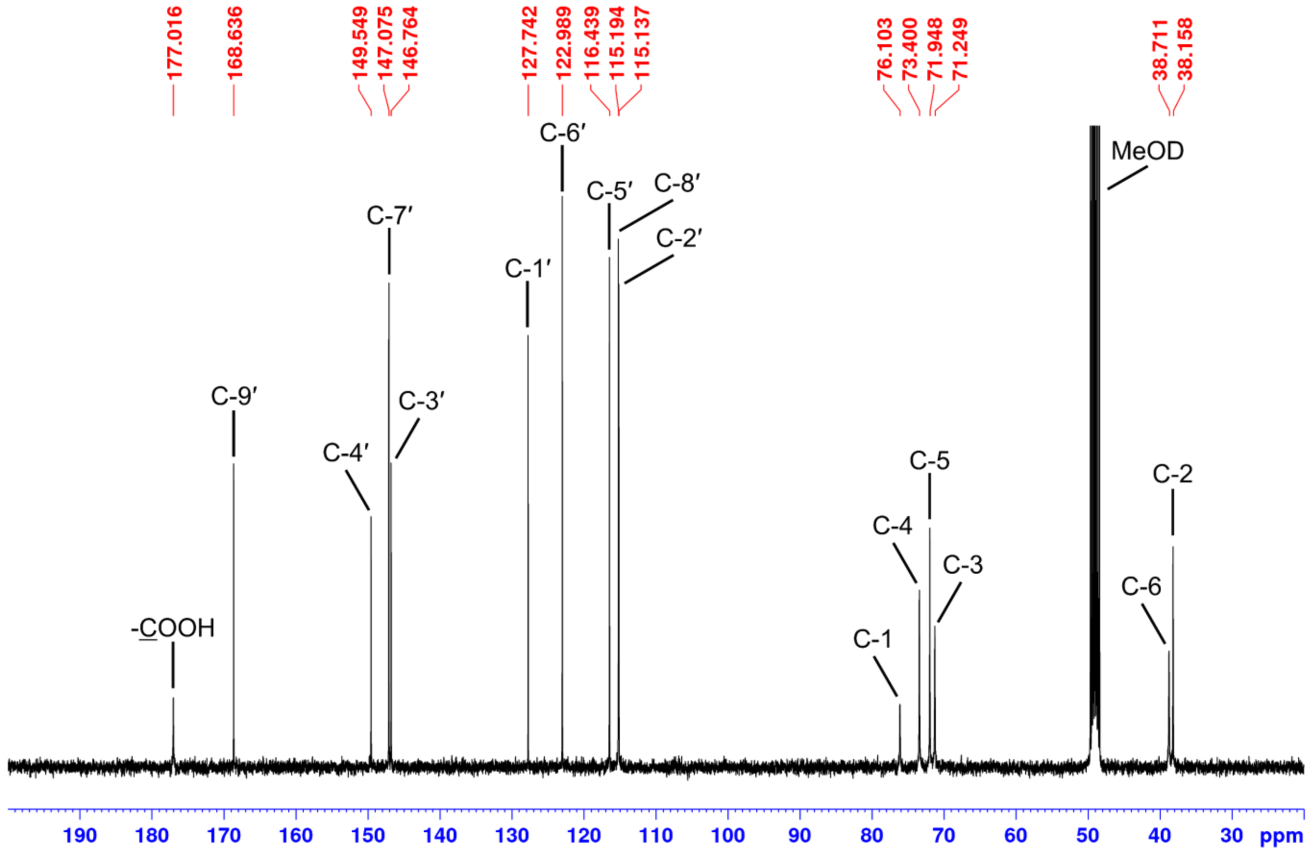

Supplementary Figure S3. (continued) NMR spectra of 5-CafQA (2).

(B) ^{13}C-NMR spectrum (100 MHz, CD_3OD)

C

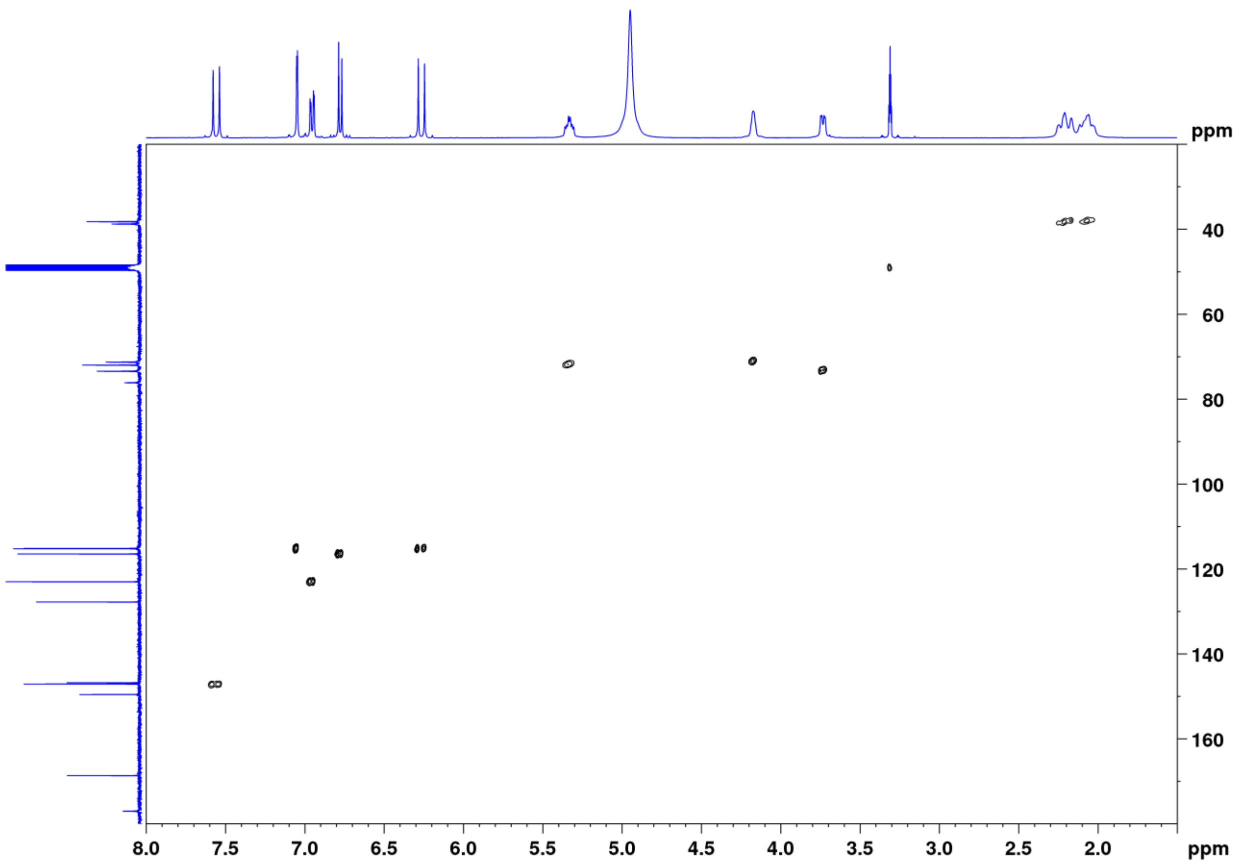

Supplementary Figure S3. (continued) NMR spectra of 5-CafQA (2).

(C) HSQC spectrum

D

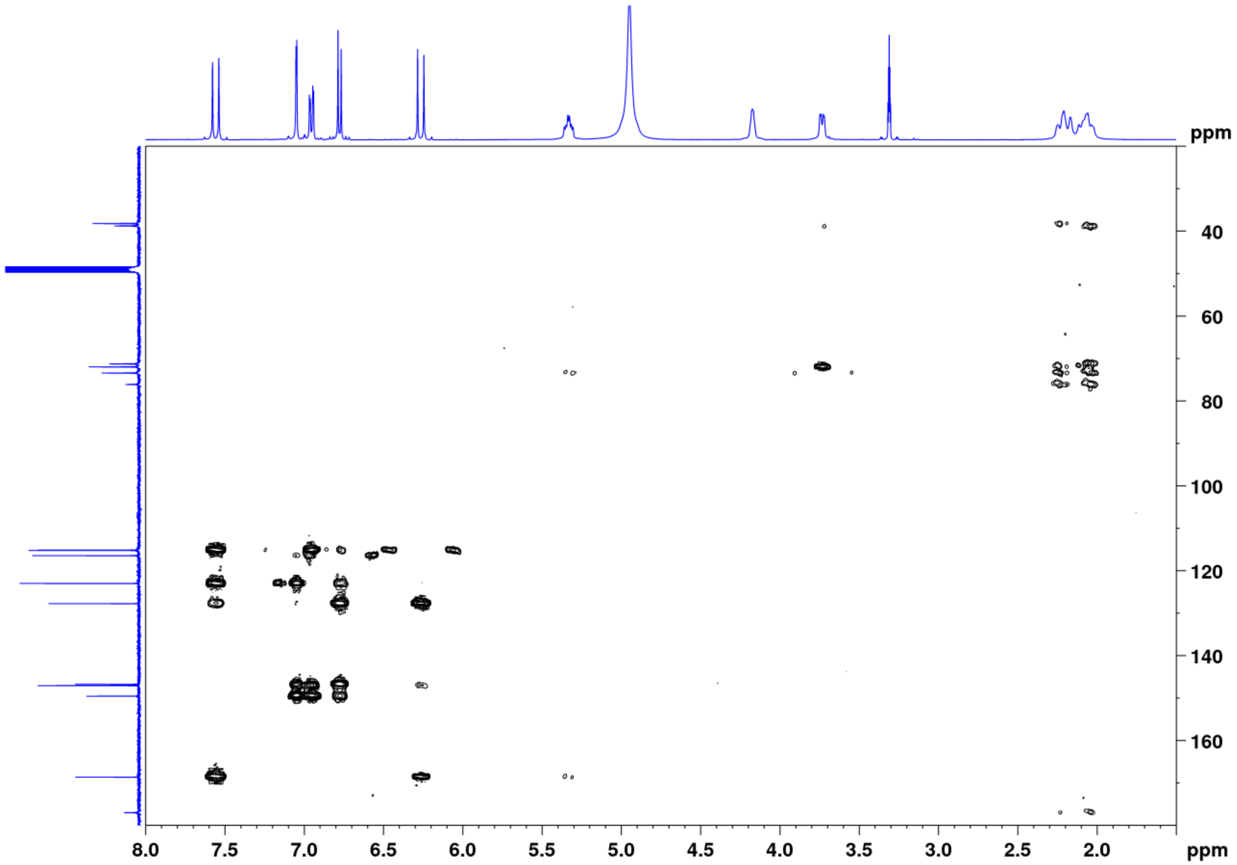

Supplementary Figure S3. (continued) NMR spectra of 5-CafQA (2).

(D) HMBC spectrum

E

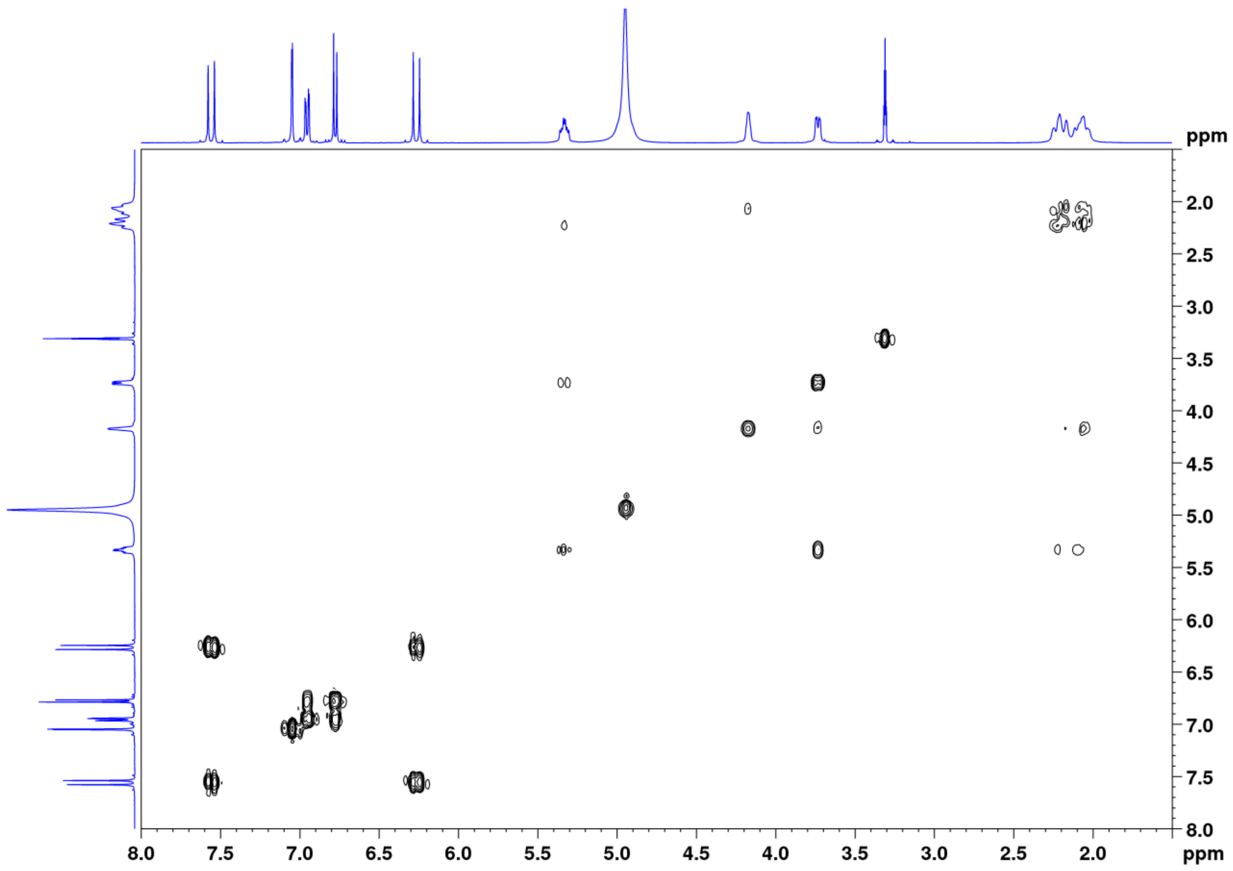

Supplementary Figure S3. (continued) NMR spectra of 5-CafQA (2).

(E) COSY spectrum

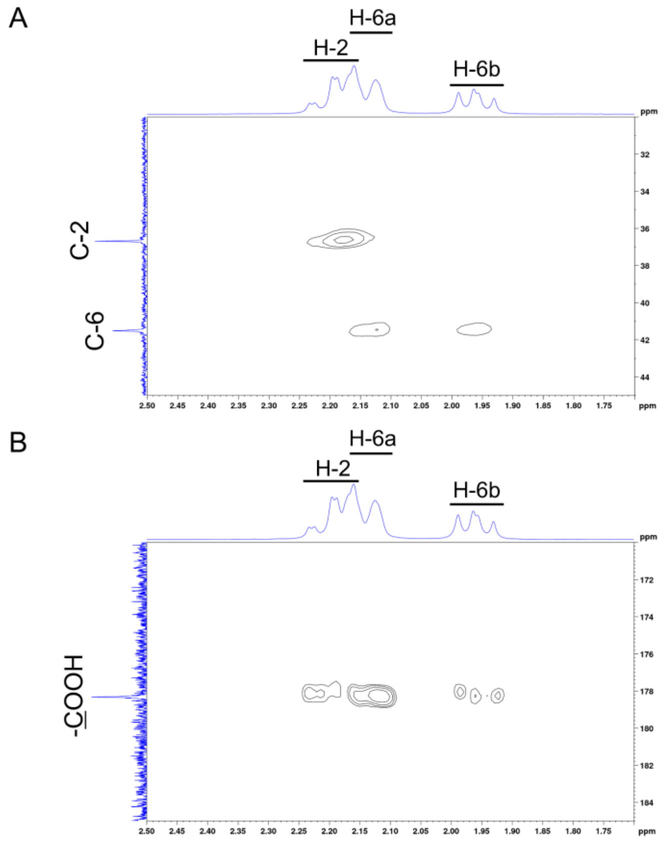

Supplementary Figure S4. Magnified HSQC (A) and HMBC (B) spectra of 3-CafQA (1). According to the HSQC spectrum (A), left side of the methylene proton signals at 2.23–2.12 ppm (H-2 and H-6a) was correlated with C-2 and the right side with C-6. Based on these observations, we confirmed that both methylene proton signals (H-2 and H-6) had correlations with carbonyl carbon signal at 178.3 ppm in the HMBC spectrum (B).

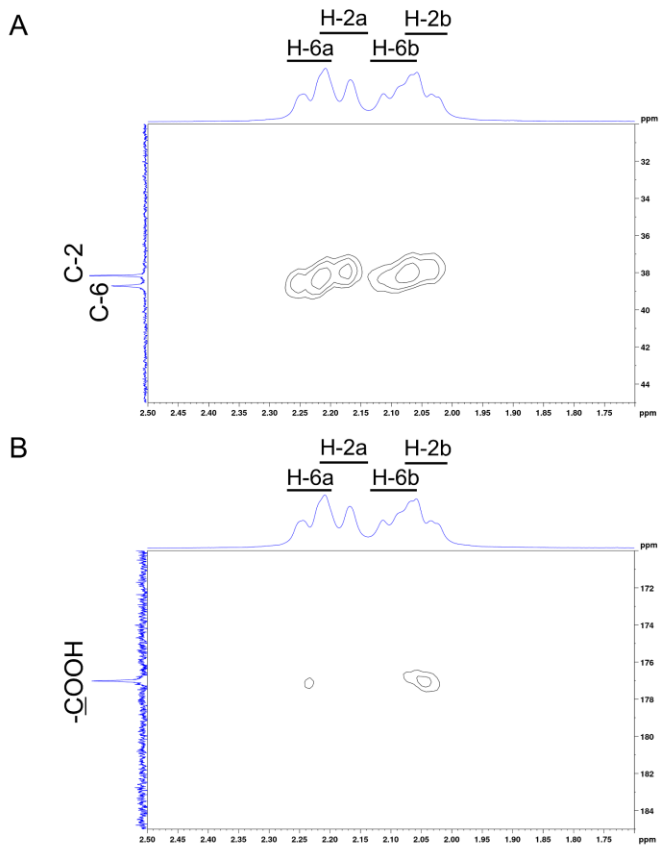

Supplementary Figure S5. Magnified HSQC (A) and HMBC (B) spectra of 5-CafQA (2). According to the HSQC spectrum (A), left side of the methylene proton signals at 2.24–2.17 ppm (H-6a and H-2a) was correlated with C-6 and the right side with C-2. The methylene proton signals at 2.11–2.03 ppm (H-6b and H-2b) also showed the similar correlations. Based on these observations, we confirmed that the both methylene proton signals (H-6a and H-2b) had correlations with carbonyl carbon signal at 177.0 ppm in the HMBC spectrum (B).

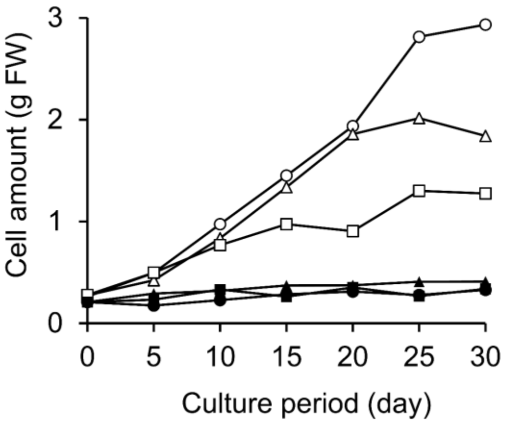

Supplementary Figure S6. Growth profiles of Pn suspension cells cultured under light or dark conditions in different basal media. Growth profiles of the suspension cells cultured in B5 (triangles), MS (circles), and White (squares) media under light (filled symbols) and dark (open symbols) conditions. Initial cell density was set to 5 % SCV (sedimented cell volume per 30-mL medium). n = 1

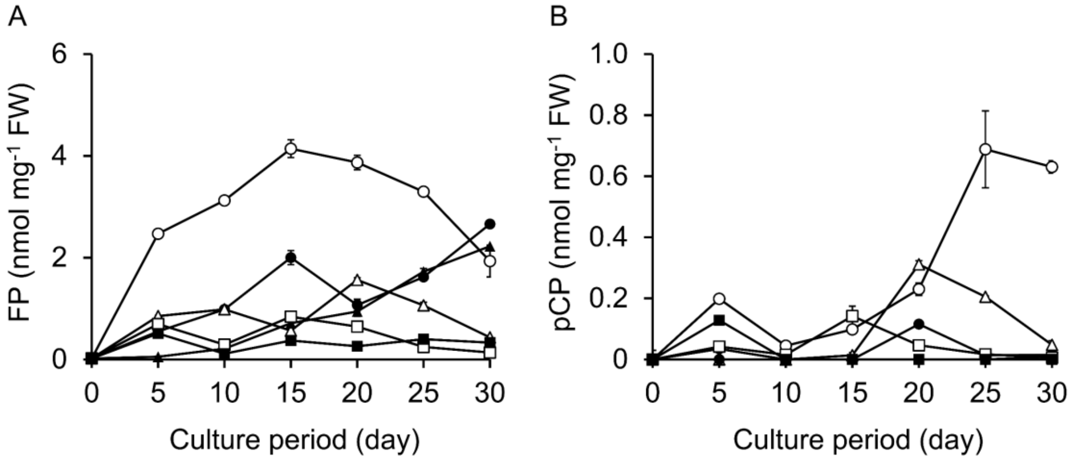

Supplementary Figure S7. Time-course of changes in FP and pCP contents in Pn suspension cells cultured under light or dark conditions in different basal media. Contents of FP (A) and pCP (B) in suspension cells cultured in B5 (triangles), MS (circles), and White (squares) media under light (filled symbols) and dark (open symbols) conditions are shown. Initial cell density was set to 5 % SCV. Data are presented as the mean ± SD (*n* = 3).

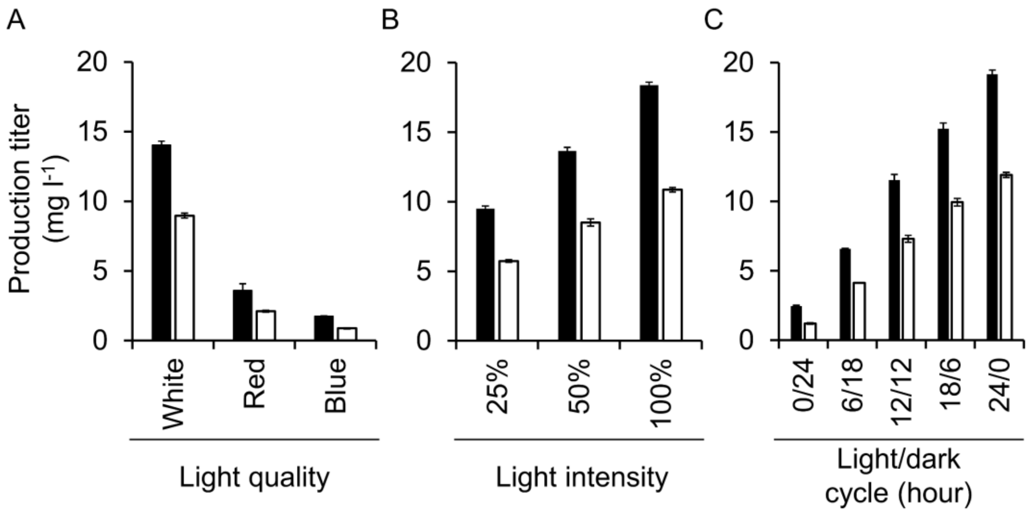

Supplementary Figure S8. Effects of light conditions on production titers of 3-CafQA (1) and 5-CafQA (2) in Pn suspension cells. Production titers of 3-CafQA (**1**) (filled bars) and 5-CafQA (**2**) (open bars) in suspension cells cultured in B5 medium for 15 days under various light conditions are shown: light quality (A), light intensity (B), and light/dark cycle (C). Initial cell density was set to 5 % SCV. Data are presented as the mean ± SD ($n = 3$).

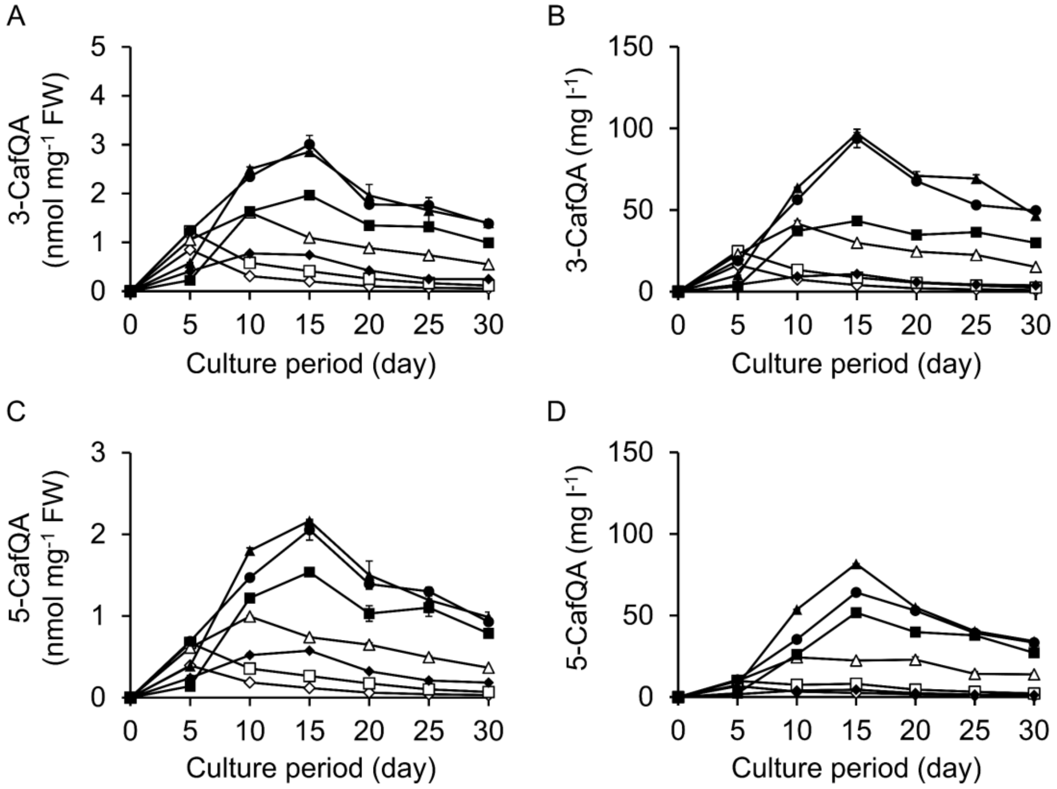

Supplementary Figure S9. Effects of sucrose strength on production of 3-CafQA (1) and 5-CafQA (2) in Pn suspension cells. Contents of 3-CafQA (**1**) and 5-CafQA (**2**) (A, C) and production titers of 3-CafQA (**1**) and 5-CafQA (**2**) (B, D) in suspension cells cultured in B5 medium with different sucrose strengths under light are shown. Sucrose strengths were as follows: ×1/8 (open diamonds), ×1/4 (open squares), ×1/2 (open triangles), ×1 (filled circles), ×2 (filled triangles), ×4 (filled squares), ×8 (filled diamonds). Initial cell density was set to 20 % SCV. Data are presented as the mean ± SD (*n* = 3).

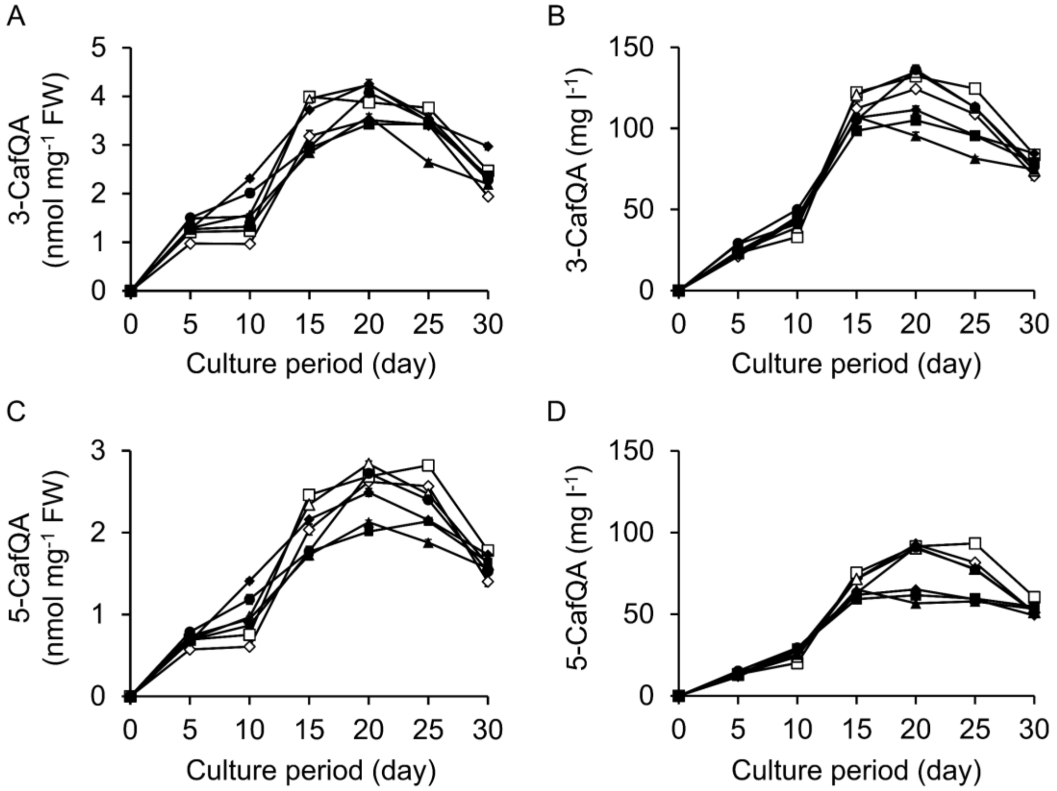

Supplementary Figure S10. Effects of stock II strength on production of 3-CafQA (1) and 5-CafQA (2) in Pn suspension cells. Contents of 3-CafQA (**1**) and 5-CafQA (**2**) (A, C) and production titers of 3-CafQA (**1**) and 5-CafQA (**2**) (B, D) in suspension cells cultured in modified B5 medium (1/4 strength of stock I) with different stock II strengths under light are shown. Stock II strengths were as follows: ×1/8 (open diamonds), ×1/4 (open squares), ×1/2 (open triangles), ×1 (filled circles), ×2 (filled triangles), ×4 (filled squares), ×8 (filled diamonds). Initial cell density was set to 20 % SCV. Data are presented as the mean ± SD ($n = 3$).
